# Geochemical characterization of millions of individual atmospheric particles entrapped in Antarctic ice across the last glacial-interglacial transition

**DOI:** 10.1038/s41598-026-45260-3

**Published:** 2026-03-30

**Authors:** Stanislav Kutuzov, John W. Olesik, Madeleine C. Lomax-Vogt, Lucas M. Carter, Gregory V. Lowry, Garret D. Bland, Jonas Wielinski, Ryan C. Sullivan, Paolo Gabrielli

**Affiliations:** 1 School of Earth Sciences, The Ohio State University, Columbus, OH 43210 USA; 2 Byrd Polar and Climate and Research Center, The Ohio State University, Columbus, OH 43210 USA; 3 Department of Chemistry and Biochemistry, The Ohio State University, Columbus, OH 43210 USA; 4 Department of Civil and Environmental Engineering, Carnegie Mellon University, Pittsburgh, PA 15213 USA; 5 Department of Chemistry, Carnegie Mellon University, Pittsburgh, PA 15213 USA; 6 Department of Mechanical Engineering, Carnegie Mellon University, Pittsburgh, PA 15213 USA; 7 Italian Glaciological Committee c/o University of Turin, Turin, Italy; 8 Present Address: The Metropolitan Water District of Southern California, La Verne, CA 91750 USA

**Keywords:** Climate sciences, Environmental sciences, Planetary science, Solid Earth sciences

## Abstract

Reconstructing the source regions of past atmospheric dust preserved in ice remains a challenge in Antarctic glaciology. Until now, different dust properties were obtained by separate techniques and could not be directly correlated at single particle level limiting the dust characterization. Here we apply a novel technique (single particle Inductively Coupled Plasma-Time of Flight Mass Spectrometry) to characterize millions of individual particles in low-volume (< 2 mL) ice samples. We analyzed more than 2,000,000 individual particles smaller than 2.5 µm in 28 discrete samples from Taylor Glacier, coastal East Antarctica, spanning 44—9 kyr BP. We show a glacial-interglacial shift in particle number and mass concentrations, as well as in the elemental and mineralogical compositions. Our observations suggest a common potential dust source area for central and coastal East Antarctica during the Last Glacial Period, followed by a transition to different dominant sources in coastal sites during the Holocene. These changes likely reflect large-scale variations in dust sources, and environmental conditions in the Southern Hemisphere. We have also identified and measured the elemental composition of thousands of volcanic particles < 2.5 µm, indicating occasional tephra deposition from one of the Victoria Land volcanoes around 14.8 kyr BP.

**Supplementary Information:**

The online version contains supplementary material available at 10.1038/s41598-026-45260-3.

## Introduction

Ice cores serve as one of the most informative archives of paleoenvironmental information. Insoluble mineral microparticles (MPs), defined here as particles larger than 0.467 µm but smaller than 2.5 µm, present in the atmosphere deposit onto the polar snowpack surface through wet and dry deposition. Subsequently, these particles become entrapped in polar ice, enabling the reconstruction of past atmospheric conditions and natural variability of the climate system at millennial and orbital timescales^1–3^.

Planetary-scale factors primarily drive natural variations in atmospheric particle abundance and properties during glacial-interglacial cycles^3^. These factors include changes in the extent and strength of dust sources; shifts in atmospheric circulation; deposition processes; and episodic events such as volcanic eruptions. Particles larger than 1 µm contribute most to the total mass of atmospheric particulate matter. However, fine (< 2.5 µm) and ultrafine (< 0.1 µm) particles dominate in number and substantially influence atmospheric chemistry, and reactivity^4,5^. As dust is transported through the atmosphere, coarse particles (> 2.5 µm) settle quickly (hours/few days) due to gravity, while finer particles can travel farther (from days to few weeks) from their sources^6^ and influence the atmospheric background and dust flux.

Previous studies of dust particles preserved in Antarctica ice have documented variations in elemental and isotopic composition, mineralogy, and potential source regions^7–9^. South America and Australia are often considered the two major dust sources between 30° and 90° S contributing over 90% of the dust deposition to Antarctica both in glacial and interglacial conditions^10^. During glacial periods, dust flux to East Antarctica predominantly from Southern South America sources increased approximately 30 to 50 times, likely due to higher dust availability at the sources and stronger atmospheric transport^2,8,11–16^. Exposed Argentine continental shelf and Patagonian glacial outwash sediments were additional potential dust sources during glacial periods^17,18^. However, during the glacial-interglacial transitions and the Holocene, local dust sources (e.g., ice-free deglaciated areas of Antarctica) and other possible sources including Australia, New Zealand, likely contributed. These potential source areas have been suggested for both the coastal East Antarctic (e.g., Talos Dome and Taylor Glacier)^8,19,20^ and central East Antarctica based on rare earth elements patterns^21,22^ and Sr-Nd isotopic composition^23^.

Modeling experiments show that under the current climate conditions South America and Australia each contribute about equally to the dust deposition in Antarctica, while there is a clear increase of South American sources during Last Glacial Maximum (LGM) due to source expansion^24,25^. Additionally, source contributions differ throughout regions in Antarctica, with higher proportion of Australian dust sources estimated for coastal regions of East Antarctica^24^. A modeling study of mineral dust cycle using ECHAM6.3-HAM2.3 model found that Australia contributed a higher proportion during the LGM, explained by changes in the precipitation patterns^10^. This contradicts previous ice core studies^8,26,27^ highlighting the need for further investigations.

Dust concentrations and size distributions in ice cores are usually measured using Coulter Counter for discrete samples and laser-based particle counters in continuous flow systems^28^. More recently, on-flow imaging microscopy was coupled with deep neural networks to perform more detailed particle size analyses^29^. Most past studies derived compositional data from bulk concentrations of major ions, such as Na^+^, Mg^2+^, SO₄^2−^, and Cl^−^, measuring meltwater samples by ion chromatography (IC). Bulk elemental concentrations were obtained by using Inductively Coupled Plasma-Mass Spectrometry (ICP-MS)^22,30,31^, Particle Induced X-ray Emissions (PIXE)^32^, or more recently by Inductively Coupled Plasma-Time of Flight Mass Spectrometry (ICP-TOFMS)^33^. Several studies also applied bulk isotopic analyses to infer dust provenance [e.g. 8,17]. While a number of studies examined mineral particles in ice cores^1–3,34^, only a few analyzed the elemental compositions of statistically significant number (>thousands) of individual particles^35^. Individual particles in ice cores have been analyzed using Scanning (or Transmission) Electron Microscopy with Energy Dispersive Spectroscopy (SEM/TEM−EDS)^36–38^ and Raman microspectroscopy^39–41^ and were generally limited to ~100–300 particles per sample. Mineralogy of the individual MPs was determined using single‐grain Raman spectroscopy in the Dome C ice core^39^ and X-ray absorption near-edge structure spectroscopy in the Talos Dome ice core^19^. These methods provide valuable structural and compositional data but require substantial measurement time and are therefore generally limited to analyzing not more than hundreds of particles per sample, restricting the ability to generate statistically representative results.

The development of single particle Inductively Coupled Plasma Time-of-Flight Mass Spectrometry (spICP-TOFMS) enabled direct analysis of number concentrations, mass-equivalent diameter distributions, and elemental compositions of individual nano- and MPs^42,43^. This technique has emerged as a powerful tool in environmental nanoscience and microscience^44–46^, with only a few applications to ice and snow samples^47–49^. It was also applied to continuous-flow analysis systems, providing high resolution records^47^. spICP-TOFMS measures a complete ICP elemental mass spectrum of each of thousands of individual particles within minutes using less than 1 mL of sample, analyzing representative compositionally distinct particle populations (< 2.5 µm) and potentially reconstructing particle sources^50–52^.

In this study, we explore variations in the dust concentration and geochemistry of millions of individual MPs (0.4–2 µm) in a horizontal ice core from Taylor Glacier located in coastal East Antarctica during the last glacial-interglacial transition from the Last Glacial Period (LGP, 115-11.7 kyr BP) to Holocene^8,53^. Unlike traditional ice cores drilled vertically from the glacier surface to bedrock, the concept of a horizontal ice core is based on the principle that ice formed in the accumulation zone is advected to the surface in the ablation zone by ice flow^54^. Therefore, by sampling surface ice along a transect perpendicular to the ice-flow direction, it is possible to retrieve a paleoclimate record. The accumulation zone of Taylor Glacier is located in the northern part of Taylor Dome, from where ice flows down Taylor Valley, where it is exposed at the surface^55^. Surface ice samples covering the deglaciation and the LGM have been used to obtain continuous records of atmospheric gases^56^, water isotopes, dust and chemistry^54^.

We analyzed number concentrations, mass-equivalent diameter distributions, and elemental compositions of millions of single insoluble mineral MPs in discrete samples by spICP-TOFMS. These samples span the last glacial-interglacial cycle (44 to 9 kyr BP) capturing the strong decline in dust deposition that occurred between the LGM (26-18 kyr BP) and the early Holocene (11.7-9 kyr BP)^8^. We investigated whether mineral MPs in this archive preserve geochemical signatures that reflect changing dust emission sources. We identified and geochemically characterized submicron mineral particles entrapped in Antarctic ice across the last climatic transition aiming to resolve mineralogical and elemental variability at the single-particle level which was previously inaccessible in bulk analysis.

## Results

### Number, number concentration, and mass equivalent diameter distributions of microparticles

Between 6700 and 823,000 individual MPs were measured in each of the Taylor Glacier ice core samples using spICP-TOFMS (Table S1). Sample volume delivered to the plasma ranged between 0.12 and 0.56 mL. In total, 2,158,100 individual particles < 2.5 µm and 330,600 particles > 0.467 µm in mass-equivalent diameter, calculated from oxide-converted elemental masses (see Methods for detailed explanations of detection limits) were identified in approximately 6.7 mL of total volume across the 28 Taylor Glacier samples.

**Fig. 1:**
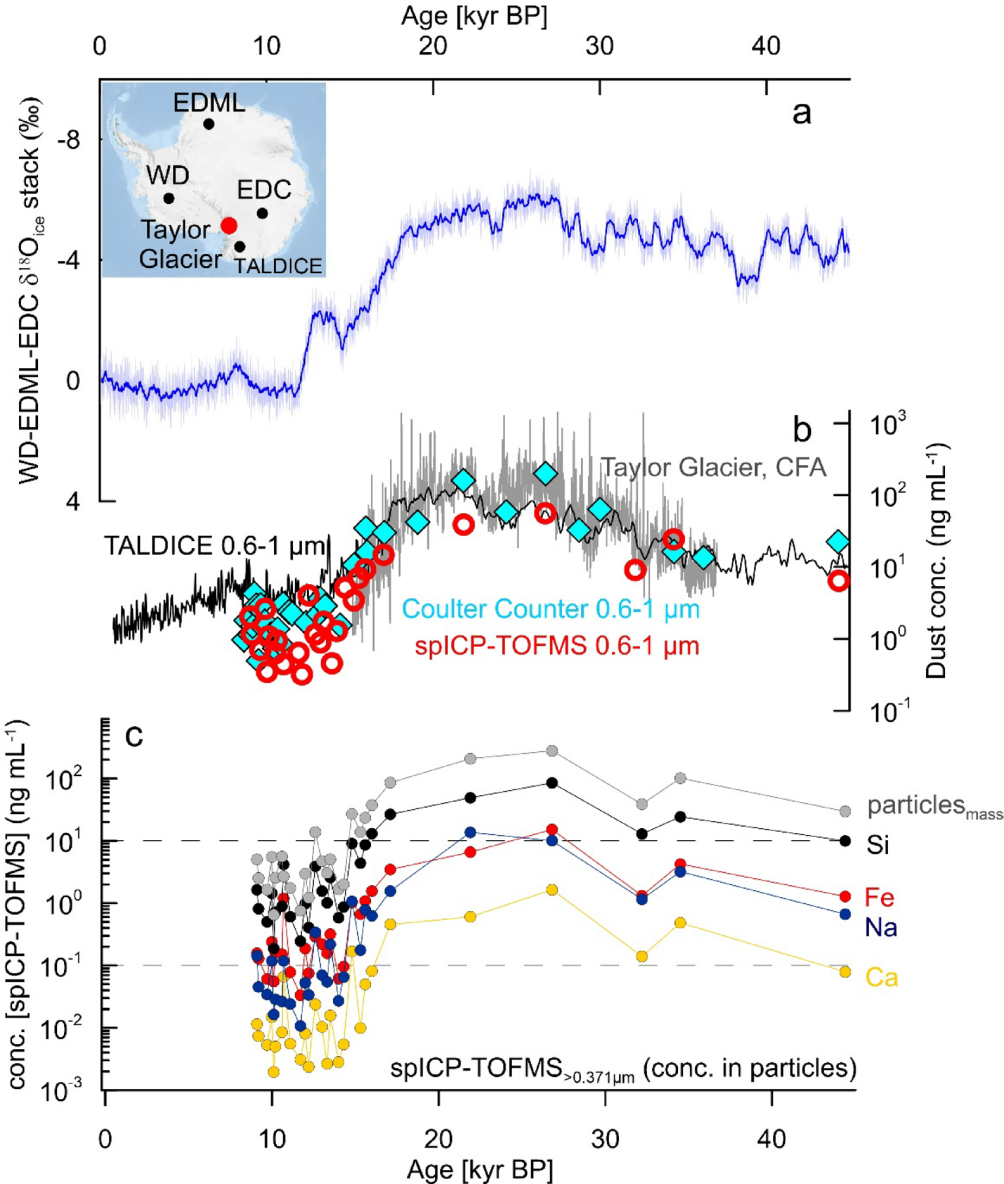
Records from the Antarctic ice cores. (**a**) Stack of the δ^18^O_ice_ records from EPICA (European Project for Ice Coring in Antarctica) Dome C (EDC), EPICA Dronning Maud Land ice core (EDML), and West Antarctic Ice Sheet (WAIS) Divide (WD) ice cores with its 2σ confidence interval^59^. (**b**) Concentrations of insoluble mineral dust: in Talos Dome (TALDICE) ice core (black line) for 0.6–1 µm particles^57,58^; in Taylor Glacier (TG) (grey line) data from Abakus laser particles counter ∼ 0.8–10 μm^54^; dust concentrations in TG samples measured for particles 0.6–1 µm using Coulter Counter (blue diamonds)^8^ and by spICP-TOFMS (red circles). (**c)** spICP-TOFMS total calculated concentrations of Si, Fe, Na and Ca in all particles larger than 0.467 μm and smaller than 2.5 μm.

Number concentrations for MPs >0.467 μm ranged from 1170 to 417,333 particles mL^−1^, while calculated total mass concentrations varied between 0.3 and 135 ng mL^−1^ (Fig. 1). Based on 5 repeated measurements of the samples, relative standard deviations typically ranged between ~0.6 and 8% for particle number concentrations, and around 10% (3.2–12.5%) for mass concentrations (sum of mass of particles mL^−1^). For low abundant elements (e.g. rare-earth elements), the associated relative standard deviations for specific element mass concentrations were larger ~15% (7.9–22.0%). Samples from the LGM (26-18 kyr BP) and between the beginning of the deglaciation and the early Holocene (18-11.7 kyrs BP) contained more particles than those from the early Holocene (11.7-9 kyr BP), reflecting higher atmospheric dust loading during the LGP and LGM compared to Holocene epoque, consistent with previous reports^3,11,54,57^. On average, LGM samples contained ~100 times more particles than early Holocene samples, with LGM/Holocene number ratios ranging between 12 and 300. Particle number concentration temporal trends were similar when measured with spICP-TOFMS or Coulter Counter (Fig. 1b). Number and mass concentrations measured by the two techniques were strongly correlated, with R^2^ values of 0.9 although the discrepancies increase for low concentration Holocene samples (Fig. S1b). Results are comparable with the previous estimates for both coastal sites in East Antarctica^58^ and EDC. Talos Dome particle concentrations in the smallest size range (0.6–1 µm) show similar deglaciation trends with some LGP differences (Fig. 1b).

Atmospheric mineral particle number size distributions can be represented by an inverse power law distribution (also called Pareto distribution) for particles with diameters between 0.1 and 10 µm^48^. Mineral particles in this size range represent the atmospheric particle background of well sorted particles arriving in Antarctica from distant sources. All particle size distributions (PSDs) of samples analyzed in this study followed the Pareto distribution (which appears as a straight line on the log-log scale) for particles with mass equivalent diameters > 0.467 µm but less than about 1 µm (Fig. S1a). PSDs can be determined by spICP-TOFMS within a sample dependent size range. Here, we report PSDs only for the sizes above the sample specific mode (see Methods). There are undoubtedly particles smaller than sample specific modes of Pareto distribution in Taylor Glacier samples, but their mass equivalent diameters cannot be accurately determined due to the high Si and Al quasi-continuous backgrounds. The particle transport efficiency from the melted sample into the ICP decreases with the size for particles larger than ~ 1 µm (Fig. S1a, d–i)^51^. Therefore, larger particles (1–2.5 µm) are underrepresented in spICP-TOFMS data explaining the different trends between the Coulter Counter and spICP-TOFMS (Fig. S1d–i).

Modern seasonally dependent central Antarctic atmospheric aerosol number PSD modes are typically 20–40 nm when the Aitken and accumulation modes considered^60^. In the LGM Antarctic ice core samples both mass and number PSDs typically follow the log-normal distribution with the mass (volume) modes around 2 µm and expected number PSD modes of 0.2–0.5 µm depending on the standard deviations^54^. Using the spICP-TOFMS we did not directly measure this mode due to the higher detection limits of the major elements (see Methods). The Pareto fit could be used as an approximation of the upper tail of the log-normal distribution when the mode cannot be fully resolved due to detection constraints. spICP-TOFMS provides valuable information about the PSD shape that can be used to assess changes in atmospheric particles over time. For example, the slope (b) of the Pareto distribution in the Taylor Glacier samples ranged from − 3 to − 3.9 (3.4±0.2), despite order-of-magnitude differences in absolute particle number concentrations of different samples which is close to typical value (b ≈ 3) for background continental atmospheric aerosols^48^. This consistency indicates that particle size distributions for these samples were governed by similar long-distance transport processes. Exceptions are the 14.8 kyr BP sample and 12.6 kyr BP sample, which contained a greater abundance of larger particles and exhibited a Pareto slope of − 2.8 (Fig. S1a) and − 2.7 respectively. The presence of larger particles in these two samples was confirmed by Coulter Counter measurements (Fig. S1e, h).

### Concentration of major elements

spICP-TOFMS also measures the element concentrations in MPs, and the total quasi-continuous background concentrations (produced by dissolved elements, particles with elemental masses too small to be identified as particles, and elemental or polyatomic ions that cause a spectral overlap) (see the Methods section for a detailed explanation). Both MPs and quasi-continuous background signal based elemental concentrations measured in Taylor Glacier samples measured by spICP-TOFMS follow the same trend as the total particle number concentrations. The LGM/Holocene elemental concentration ratios varied for different elements, with the ratios of background concentrations always being smaller than the ratios of elemental concentrations in particles. On average, the concentrations of major elements calculated from the quasi-continuous background signal between 26 to 18 kyr BP were 20 (min–max 8–36) times higher than during the early Holocene (9-11.7 ka BP). However, the total concentration of these elements in samples due to the MPs was, on average, 76 (37–150) times higher, with large sample to sample variations (Fig. 1c). This suggests that the input of coarser-insoluble particles changed significantly more than the input of ultrafine-soluble particles/elements during the glacial-to-interglacial transition.

**Fig. 2:**
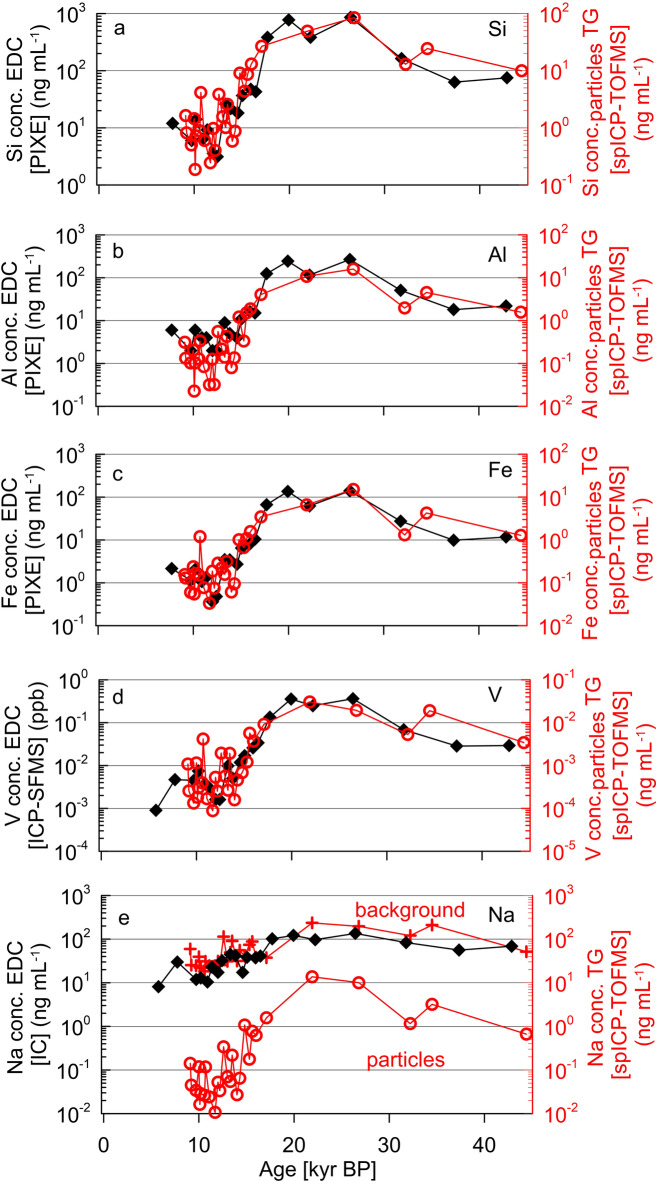
Concentrations of elements in insoluble particles in Antarctic ice. Taylor Glacier samples were measured by spICP-TOFMS (red) while EDC ice core samples by PIXE (bulk), ICP-SFMS (acidified bulk), and IC (black)^61^. Measurements of the (**a**) Si, (**b**) Al, and (**c**) Fe, (**d**) V, and (**e**) Na are shown. Note the different scales used for the left vs. right axes for a-d). Concentrations of Na in spICP-TOFMS background are also shown (**e**) (red crosses).

Among the major elements in water-insoluble MPs, Na, Mg, Mn, Al, and Ca showed the greatest variations in total mass concentrations over time, with LGM-to-Holocene ratios > 70. In contrast, Si, Ti and Fe mass concentration due to MPs during LGM were < 50 times greater than during the Holocene. Interestingly, the measured LGM-to-Holocene ratios from these elements in the Taylor Glacier ice cores match the EDC ice core ratios measured by PIXE^61^ (Fig. 2a–c). Absolute total concentrations of elements in particulate matter measured by spICP-TOFMS were 10 times lower than the corresponding total values obtained by PIXE (Fig. 2a–c), and ICP-SFMS (Fig. 2d) as the submicron particles in Antarctic ice samples comprise only about 10% of the total mass of dust particles measured by Coulter Counter (this work). The similarity in bulk concentration LGM/Holocene ratios between EDC and TG suggests there were similar changes in fine and ultrafine dust particle composition in both interior and coastal sites. Dissolved Na bulk concentrations measured by IC for EDC samples were similar to the spICP-TOFMS measurement of Na based on the quasi-continuum background signals in Taylor Glacier ice and changed by less than a factor of 10 from 10 kyr BP to 20 kyr BP. In contrast, the Na concentration due to Na in individual water-insoluble particles measured by spICP-TOFMS shows a large increase in LGM samples (Fig. 2e).

In terms of the relative contribution of major elements to particles chemical composition, more than 99% of the total (cumulative) particulate mass detected in all Taylor Glacier samples was due to major elements Na, Mg, Al, Si, Ca, Ti, and Fe. Here, the reported concentrations of major elements are converted and expressed as oxides based on stoichiometric molar ratios (as is commonly done with bulk X-ray fluorescence measurements of geological samples), as spICP-TOFMS does not directly measure oxygen; this enables comparison with results from other analytical techniques. In general total sample composition resembles the signature of the upper continental crust (UCC)^62^, although, on average, it is depleted in Ca (CaO= 0.5 (0.1–0.9) wt% vs 3.6 wt% in UCC), Mg (MgO=1 (0.3–2.8) wt% vs 2.5 wt%) and Al (Al_2_O_3_ = 9.8 (5.4–13.5) vs 15.8 wt %) and enriched in Fe (Fe_2_O_3_ = 7.7 (4–14.6) wt% vs 5.2 wt%), Ti (TiO_2_ = 1 (0.4–1.8) wt% vs 0.7 wt%) and Si (SiO_2_ = 76.5 (70.3–82.6) wt% vs 68.5 wt%). The depletion in CaO and enrichment in Fe and Ti was also noted in the Talos Dome ice core and was attributed to the chemical weathering of local sedimentary rocks acting as dust sources within East Antarctica^19^. The overall composition of particulate matter, calculated from the cumulated masses of all detected elements, remained broadly consistent across the measured samples (Fig. S2). However, LGM samples contained higher proportions of Na and Mg (Na_2_O_LGM_ = 5.7 (5.3–7.5) wt% and MgO_LGM_ = 2 (1.6–2.8) wt%) compared to early Holocene (Na_2_O _Holocene_ = 2.7 (1.4–4.1) wt% and MgO _Holocene_ = 1 (0.8–2.1) wt% for the LGM samples). Holocene samples also tended to be relatively enriched in Fe compared to LGM samples (Fe_2_O_3LGM_ = 7.1 (5.3–8.5) wt%; Fe_2_O_3Holocene_ = 9 (4.8–14.6) wt%) (Fig. S2). There was also a high variability in the Fe contribution to the total mass of insoluble particles in the Holocene, from 4.8 wt% up to 14.6 wt%. Increased Fe contribution to the total dust flux during the Holocene were also observed in Talos Dome ice core^63^. During the LGM the proportion of MnO (MnO_LGM_ = 0.15 (0.1–0.2) wt%) was slightly higher than in the UCC (MnO_UCC_ = 0.1 wt%) and then decreased to 0.05 (0.03–0.08) wt% in the 9–17 kyr BP period with the exception of the 14.8 kyr BP sample which had a 3.5 times higher proportion of MnO (0.17 wt%) than the average over this period.

### Elemental composition of individual particles

**Fig. 3:**
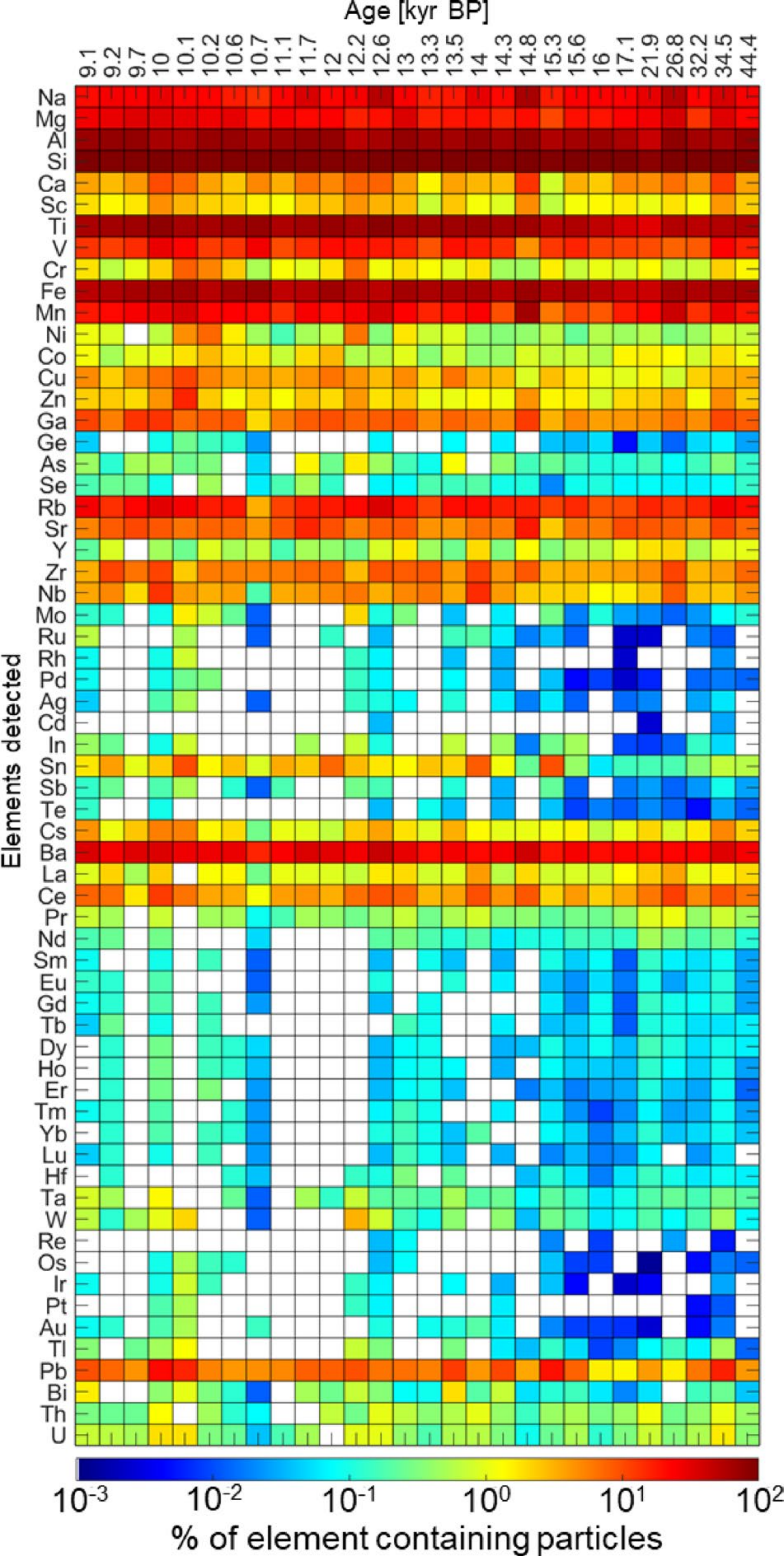
Heatmap showing the percentage of individual particles containing detectable amounts of each element measured by spICP-TOFMS in Taylor Glacier ice core samples**.** Colors indicate the percentage of particles in each sample that contains a given element displayed on a log- scale (white indicates non-detectable concentrations). The y-axis: elements from major (e.g., Na, Mg, Al, Si) to trace and rare earth elements (REEs), the x-axis represents sample age.

A unique advantage of analyzing ice core samples using spICP-TOFMS is the ability to measure a complete ICP mass spectrum in each of the detected individual atmospheric dust particles. We found that the percentage of particles containing detectable amounts each element was generally consistent through time with larger variations for the Holocene samples (Fig. 3). On average in 95.8±2.5(STD)% of all measured particles Si, 69.7 ± 7.6% Al, 63.1 ± 10.5% Ti, 58.7 ± 7.8% Fe, 27.2 ± 11.1% Na, 23.9 ± 7.4% Mg, 22.8 ± 11.5% Mn and 5 ± 2.9% Ca were detected (Fig. 3). Other common elements in particles include Ba, which was detected in 28% of the particles, Rb (18%), V (15%), and Pb (8%). We did not observe specific temporal trends in the percentage of particles containing major elements. However, certain major elements and their combinations were present in higher proportions of particles in several samples. Greater than 50% of the particles in the 12.6 kyr BP, 14.8 kyr BP and 26.9 kyr BP samples contained Na, and 64.7% of the particles in the 14.8 kyr BP sample contained Mn.

In some particles only one element was detected. Particles may contain other elements at a mass too small to be detected or elements with such a high spectral background that cannot be measured by techniques based on ICP-MS (e.g., O, H, etc.) Approximately 10% of all analyzed particles >0.467 µm contained only a single detected element. About 98 % single-element particles contain Si. Around 1.7% of single-element particles contain Fe. About 70% of all detected particles contained various combinations of 2 to 6 elements. The most common two-element combination was Al and Si, on average comprising 38% of the two-element combinations in samples, likely reflecting the abundance of aluminosilicate minerals (e.g., feldspars, clays). Other frequent two-element combinations include Si with Ti and Si with Na. Three-element combinations included Al–Si–Ti, Al–Si–Fe, and Na–Al–Si, indicating potentially more complex mineralogy, such as iron-bearing aluminosilicates or weathered lithogenic particles. Particles containing the combination of Mg–Al–Si–Ti–Fe were present in all samples and is consistent with complex silicate minerals containing Fe and Mg, such as amphiboles and pyroxenes. Particles containing one to ten elements account for approximately 96% of the total particle population. Thirty or more elements were detected in only 13 particles across all samples. In one exceptional particle of 1.6 µm in diameter from the 34.5 kyr BP sample 62 elements were detected.

**Fig. 4:**
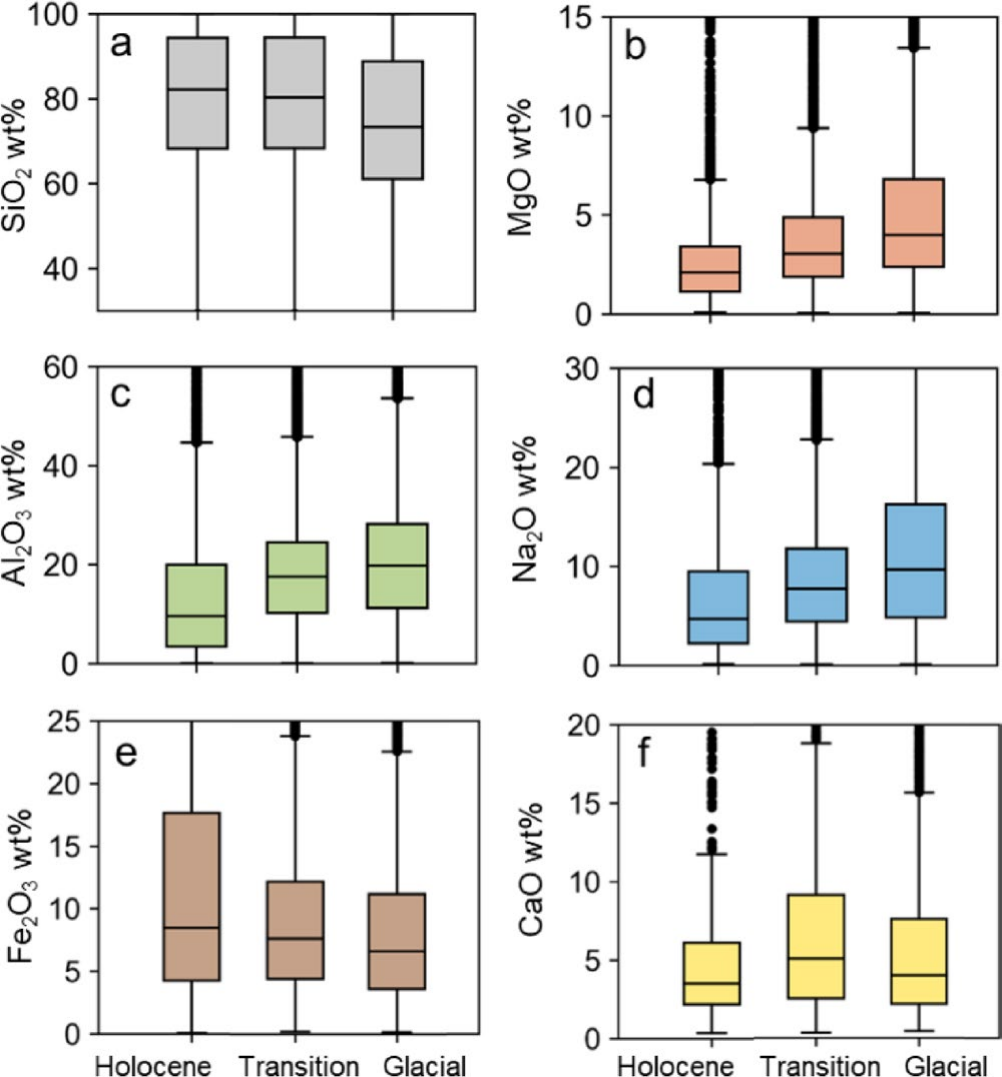
Boxplots of normalized oxide mass fractions (wt%) for selected major oxides in individual particles grouped by age category**.** (**a**) SiO_2_, (**b**) MgO, (**c**) Al_2_O_3_, (**d**) Na_2_O, (**e**) Fe_2_O_3_, (**f**) CaO. Three age bins represent the early Holocene (9.1–11.7 kyr BP), the transitional period (10.0–17.1 kyr BP), and a portion of the glacial period (21.9–34.5 kyr BP). Each box shows the interquartile range (IQR), with medians as horizontal lines and whiskers extending to 1.5*IQR. The distributions represent aggregated particle data across multiple samples per age bin.

The distributions of percent mass of Si within individual atmospheric MPs (expressed as oxide) remain relatively constant (medians of 80%) in samples younger than 18 kyr BP (Fig. 4a). MPs in older samples tend to show higher fractions of Mg (Fig. 4b), Al (Fig. 4c), and Ca (Fig. 4f), and lower fractions of Si (Fig. 4a). We observe that the median % mass of Si within individual MPs during LGM decreases with a median value of 73%. However, the oldest 44.4 kyr BP sample median value was 79% which is similar to Holocene values (Fig. S4). This is associated with an increase in the % mass of Mg (Fig. 4b) Al (Fig. 4c), and Na (Fig. 4d) within individual MPs during the glacial period with distributions centered around 19.8%, 9.7%, and 4.0% (median), respectively, decreasing during the Holocene to 9.6%, 4.7% and 2.1%. In contrast, the proportion of particles enriched in Fe (Fig. 4e) increases during the Holocene, with the upper interquartile range reaching ~17 wt% compared to ~ 12 wt% in LGM samples. Pairwise Mann–Whitney U tests indicate statistically significant differences in oxide composition for all pairwise comparisons among the Holocene, Transition, and LGM periods (Holocene-Transition, Holocene-LGM, and Transition-LGM; p < 0.001), with two exceptions: SiO_2_ shows no significant difference between the Holocene and Transition periods (p = 0.402), and CaO shows a weaker but still significant difference between the Holocene and LGM periods (p = 0.0053).

### Changes in mineralogy of individual particles

**Fig. 5:**
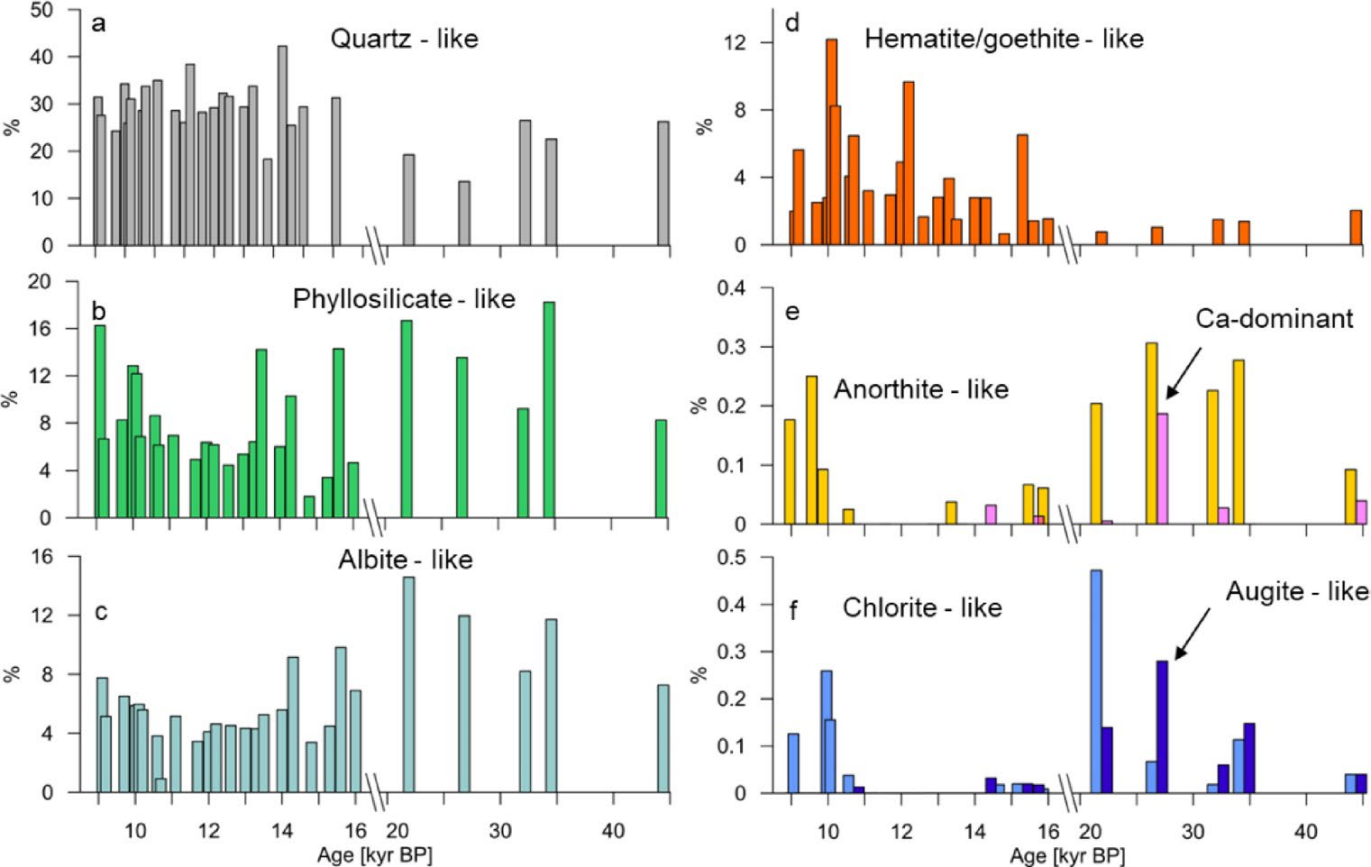
Glacial/interglacial variability in the relative abundance of mineral-like particle groups identified in Taylor Glacier samples using elemental composition-based classification. Panels show percentages of particles out of the total number of particles in each sample classified as: (**a**) quartz-like, (**b**) phyllosilicate-like, (**c**) albite-like, (**d**) hematite-like, (**e**) anorthite-like and Ca-dominant, and (**f**) chlorite-like and augite-like, plotted against sample age. The age axis is broken between 17.5–21 kyr BP.

We classified 330,600 particles > 0.467 µm from 28 samples into 14 mineral groups (see definitions in Table S5. The percentage of individual particles classified as quartz-like (Si-dominant (> 90% mass)) MPs varied between 24 and 42% during the 9–18 kyr BP except for the 14.8 kyr BP sample (18%) representing, on average, 30% of the particles. Quartz-like particles decreased in LGM samples (20%) (Fig. 5a). Albite-like and phyllosilicate-like particles, on the other hand, were more abundant in the LGM samples (%) compared to the early Holocene (%) (Fig. 5b, c). About 12% of particles during the LGM were classified as Albite-like compared to 5% after 18 kyr BP. This is consistent with a lower contribution of Si within individual particles and a larger contribution of Al, Na, and Mg during the glacial period (as shown in Fig. 4). Anorthite-like particles as well as Ca-dominant (> 60% of mass) particles were present in all samples more than 18 kyr BP while they were absent in most of the younger samples as well as chlorite-like particles. We also detected a small number of diopside particles in glacial samples. Hematite/goethite-like particles were abundant in the Holocene samples accounting for 4% particles on average (1–12%) while representing only 1% of the particles during LGM (Fig. 5). We detected an increase in the percentage of particles classified as anorthite-like, augite-like, and diopside-like in LGM samples. This increase can also be seen in the ternary diagrams for the Holocene and LGM representing pyroxene composition (Fig. S5). There was a larger number of particles containing a larger percentage of MgO in LGM samples, consistent with the augite and diopside elemental composition. These results demonstrate the potential of spICP-TOFMS for investigating the mineralogy of sub-micron particles in samples with low particle concentrations.

### Volcanic particles

The 14.8 kyr BP sample seems unique as it contained a notably higher proportion of large particles than any other measured sample, as indicated by both spICP-TOFMS (Fig. S1a) and Coulter Counter measurements (Fig. S1e, h). It also had a high percentage (%) of Mn-bearing particles containing relatively elevated masses (fg) of Mn, Sr, and Nb. Particles bearing detectable amounts of the six-element combination of Na, Al, Si, Ti, Mn, and Fe were more abundant (35%) in the 14.8 kyr BP sample (Fig. S3d). In fact, in most samples, particles containing detectable amounts of all six of these elements are less common, accounting for only 0.4–8.4% of the total population, and 2 or 3 element associations dominate. However, in two other samples: 12.6 kyr BP (13.9%), 26.9 kyr BP (16.8%), these complex particles were also relatively more abundant (Fig. S3d). The 14.8 kyr BP sample also displays lower variability in elemental composition of individual particles compared to other samples (Fig. S4). These findings suggest a distinctly different source of the particles contained in this sample. Larger particles sizes and unusual elemental composition may point to the possible presence of abundant volcanic particles in the 14.8 kyr BP sample.

**Fig. 6:**
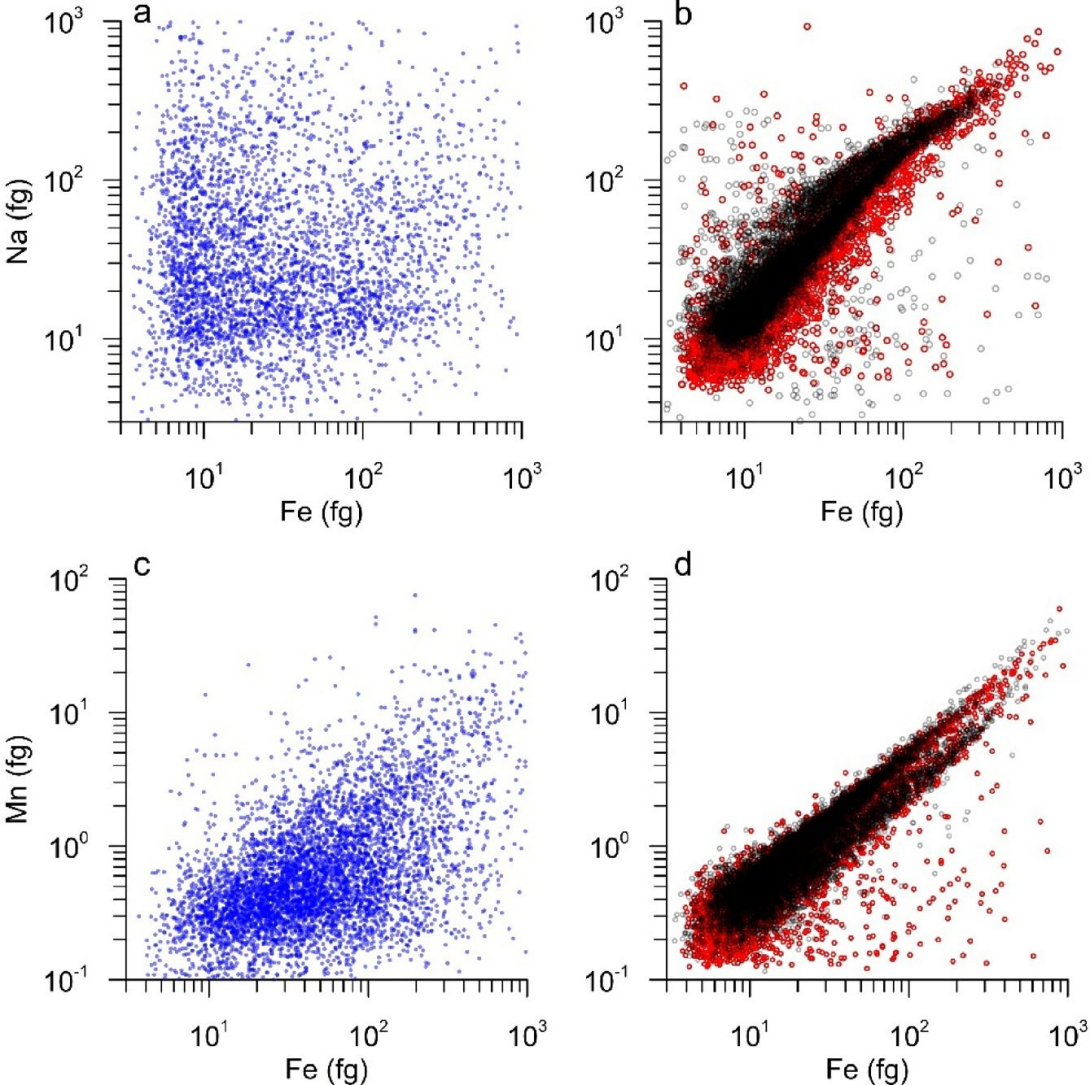
Correlations of elements in individual particles. Scatter plots show relationships between Fe and Na (**a**, **b**), Fe and Mn (**c**, **d**) in fg measured by spICP-TOFMS. Blue circles represent all particles from all Taylor Glacier samples except the 14.8 kyr BP sample, red circles show particles from the 14.8 kyr BP Taylor Glacier sample, and black circles indicate 81003G Erebus glass standard.

To test this hypothesis, we compared this sample with a reference sample Erebus volcano glass (81003G)^60^, which was ground down to powder in our lab and measured by spICP-TOFMS. The Erebus glass particles revealed strong correlation (R^2^ = 0.75–0.93) among most major elements (Na, Mg, Si, Ti, Mn, Fe) as well as several trace elements (e.g. Rb, Sr, Ba). Correlations further improve when only particles with detected major elements common for volcanic ash particles^61^ are included (Fig. S6). Remarkably similar relationships were also observed in the 14.8 kyr BP sample, although this Taylor Glacier sample is slightly enriched in Si and depleted in Ti when compared to the 81003G reference (Fig. S6). Approximately 60% of the measured particles in the 81003G standard contained all six major elements. In addition to the six-element combination discussed above, the seven-element combination including Ca was detected in 11% of the standard particles, comparable to 10.5% of the particles in the 14.8 kyr BP sample (Fig. S3e). Correlations among most major elements are also present in the 12.6 and 26.9 kyr BP samples. However, these relationships are generally weaker and some are absent (e.g., Na–Mg, Na–Ba and Ti–Ba) compared to 14.8 kyr BP sample. In the remaining samples, isolated correlations between major elements are also observed. (Fig. 6, Fig. S6).

**Fig. 7:**
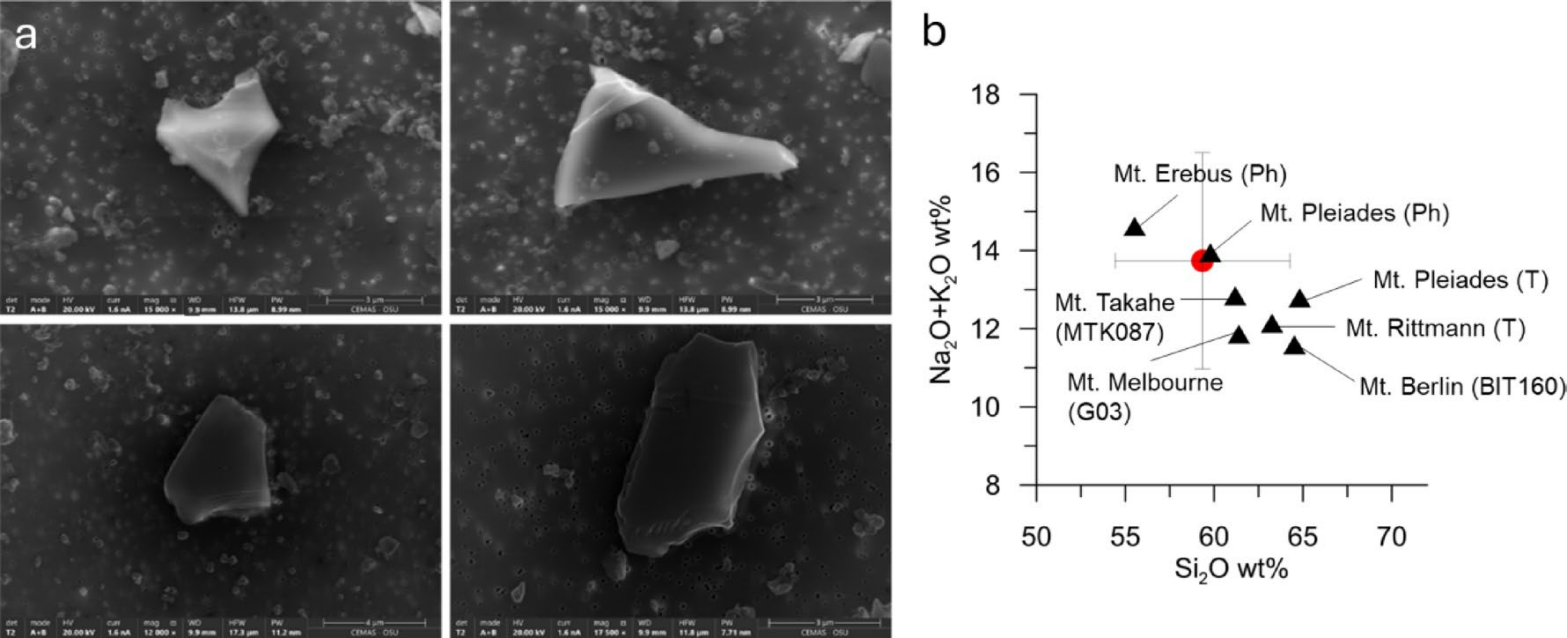
SEM images of particles from the 14.8 kyr BP sample (**a**) SEM images reveal irregular, angular morphologies and rough surface textures characteristic of volcanic glass shards. Total alkalis versus silica diagram (**b**). Average composition of 15 volcanic glass shards measured by SEM–EDS found in the 14.8 kyr BP sample is shown with the red circle and standard deviation. T, trachyte; Ph, phonolite. Also shown are average data for rocks and tephra from: Mt. Melbourne^64^, The Pleiades and Mt. Rittmann^65,66^, Mt. Erebus^67^, and Mt. Berlin and Mt. Takahe^68,69^.

To corroborate this interpretation, we also conducted additional SEM-EDS measurements on particles from the 14.8 kyr BP sample. Abundant large particles (> 5 µm) were observed. Fifteen of the 38 particles analyzed appear to be volcanic glass shards based on both morphology (smooth, sharp-edged, angular, curved) and geochemical composition (Fig. 7). Measured glass shards show an alkaline character which, together with the large size of these particles, suggest that they originate from the nearby Antarctic volcanoes known to produce alkaline magmas (phonolite, trachyte) (Fig. 7b). While our SEM-EDS results are semi-quantitative, the method has significant limitations such as underestimating Na_2_O, overestimating Al_2_O_3_ and being less sensitive to elements present at low concentrations or near detection limits^62^.

## Discussion

Overall, mass equivalent size distributions of dust particles entrapped in the Taylor Glacier samples measured with Coulter Counter in this study (Fig. S1d–i) are consistent with previous results from other coastal ice cores (TALDICE, Taylor Dome). Notably, the early Holocene samples contain poorly sorted particles, including a large fraction in the 5–10 µm range, in contrast to well sorted particles with mode around 2 µm observed during the LGM^58,70^.

Previous measurements indicate that changes in snow accumulation during the glacial interglacial transition do not explain the differences in concentration levels of dust particles reaching Antarctica^3^. We calculated the dust flux for < 2.5 µm particles measured by spICP-TOFMS using calculated snow accumulation values^8,71^. The average LGM/Holocene particle flux ratio measured by spICP-TOFMS (0.467–2.5 µm) is 25, although highly variable from sample to sample (range 7–44) and close to ratios obtained in the EDC core^72^. In contrast, the Taylor Glacier dust flux calculated using Coulter Counter data (0.6–18 µm) results in an LGM-to-Holocene flux ratio of ~ 4 only which is similar to results obtained in Talos Dome^58^, likely due to the input of larger particles of local origin. Because Holocene samples typically contain a higher proportion of 1–2.5 µm particles, they may be more strongly affected by the underrepresentation of particles > 1 µm in spICP-TOFMS data. Indeed, when only particles 0.6-1 µm for both methods they produce similar dust flux ratio of ~ 30. This indicates that spICP-TOFMS could be used to assess background aerosol particles (< 1 µm) concentration changes in ice cores. This method also appears less prone to contamination compared to Coulter Counter measurements as it does not involve any addition of reagents (electrolyte) while particles are detected directly from sample aliquots.

Changes in the terrestrial input during the glacial/interglacial transition affected both soluble and insoluble species^30^. During cold stages, continental salts contained in dust particles increased by a factor of 30, compared to a five-fold increase in sea salt concentrations in Central Antarctica^30^. EPICA nss-Ca concentrations and particles number and mass concentrations changed by a factor of ~ 50, from LGM to the Holocene^73,74^. Comparison of the total element concentrations due to insoluble particles measured by spICP-TOFMS to the concentrations calculated from the spCP-TOFMS quasi-continuous signal background supports the interpretation that the input of insoluble terrestrial particles increased more during the LGM when compared to the input of soluble species.

The relative contribution of major elements to particles composition in Taylor Glacier samples, while it is close to the UCC, it differs for CaO, which is depleted relatively to UCC (0.3–1 wt% vs 3.6 wt% in UCC) (Fig. S2). This result is in line with the analysis of the fraction of CaO in insoluble dust measured by PIXE in the EDC ice core, which also showed a lower proportion of CaO when compared to UCC (1.55 ± 0.62 wt%)^32^. The depletion likely arises from the transformation of carbonates and bicarbonates during atmospheric transport to Antarctica, as they react with acidic species (HNO_3_, H_2_SO_4_) to form soluble calcium salts. We also found that the wt% of Na_2_O in particles during the 9-18 kyr BP period (3.1%) is close to the ~ 3.3% abundance in the UCC. However, Na_2_O wt% was higher during the LGM (~ 6%), likely reflecting the input of mineral particles from a different source region. MgO is depleted in the Holocene particles, averaging 1.1% (0.7–2.1%), whereas the fraction in the LGM particles MgO (2.2%) is closer to the UCC value (2.5%).

Overall, we observe similar trends also in the mineralogy of particles collected from Taylor Glacier and East Antarctica, though some notable differences exist. Paleari et al.,^39^ reported for Central East Antarctica that quartz and feldspars were the dominant components of dust during the glacial period, together accounting for 40–60% of all classified particles based on single-grain Raman spectroscopy. In their study, quartz made up 15.2–22.8% of particles during the early Holocene. This range is similar during the LGM (13–20.6%). In our dataset, quartz-like particles also represent a significant fraction, averaging ~ 30% during the early Holocene and decreasing to 13.5–19.2% in LGM samples. The higher proportion of quartz-like particles during the early Holocene suggests a greater contribution from well-weathered continental dust. In contrast, the lower quartz fraction during the LGM implies an increased contribution of less weathered material, such as glaciogenic sediments and volcanic rocks produced by intensified physical erosion and expanded ice margins in Patagonia.

The total relative contribution of feldspars cannot be directly compared, as our results do not include potassium (K) due to a severe spectral overlap with ArH^+^. This may explain the high percentage (48%) of unclassified particles, which have elemental ratios that do not match any defined category. Most of these particles may be feldspars or volcanic minerals (such as sanidine and volcanic glass shards), as well as K-bearing clays, all of which have previously been detected in Antarctic ice cores (e.g.^38^).

The abundance and temporal trends of albite-like particles in the Taylor Glacier samples are consistent with other observations from East Antarctica^39^. In both datasets, the relative contribution of albite decreases from the LGM to the Holocene, from 11 to 6.9% in East Antarctica and from 13.3 to 5.5% in Taylor Glacier. A second notable trend is the increasing proportion of Fe-rich particles in early Holocene samples. Although hematite and goethite cannot be distinguished from each other in the Taylor Glacier samples, the overall trend previously reported^39^ for hematite is reflected in the abundance of Fe-rich (hematite-like) particles, which rises from approximately 1% during the LGM to about 4% in younger samples, with values ranging from 1 to 12%.

Fe associated with windblown mineral dust, including MPs and nanoparticles (NPs), is an important micronutrient for phytoplankton in the oceans, where a significant fraction of the “dissolved” (< 0.4 µm) Fe is actually composed of NPs. Fe oxide NPs can support the growth of phytoplankton after photo-reduction or thermal dissolution. It has been suggested that the strong increase in atmospheric CO_2_ during the last deglaciation is associated with a decrease in the aeolian bioavailable Fe supply to the Southern Ocean^75^. Fe fertilization experiments have shown that the resulting increase in plankton population leads to a pronounced increase in CO_2_ drawdown rate^76^. There is also the likely increased production of dimethyl sulfide, which escapes into the atmosphere, where it is oxidized to sulfuric and methane sulfonic acids. These condensable gases provide condensation nuclei for marine stratus clouds, leading to increased albedo and lower surface temperatures^77^. Taken together, the mineralogical changes observed in the Taylor Glacier record suggest that the deglacial transition was accompanied not only by a reduction in total dust flux but also by a shift in dust composition and potential bioavailability. The increase in Fe-rich MPs during the early Holocene implies a relative enrichment in iron oxide phases that may represent a more soluble or reactive fraction of aeolian Fe delivered to the Southern Ocean and may have influenced ocean productivity and associated feedbacks within the climate system during the last deglaciation. Further work is required to quantify the solubility and bioavailability of these particles.

We did not detect any Ca-rich particles in samples younger than 14 kyr BP, with a maximum of 14 particles (0.2%) detected in the 26.8 kyr BP sample. This supports previous findings that Ca-rich minerals (like aragonite) were present during the LGM but are absent in Holocene samples^39^. However, the abundance of Ca-rich particles in our dataset is lower (< 0.5%) than that reported in earlier studies (e.g. 3–14% of calcite particles and 8–21% of aragonite particles were detected in LGM EDC ice core samples^39^), likely due to the analytical issues associated with detecting calcium by spICP-TOFMS (see Methods).

A key difference between the EDC and Taylor Glacier datasets lies in the trends observed for pyroxenes (augite, pigeonite, diopside). The input of augitic clinopyroxene particles increased in Holocene samples of the Dome C ice core compared to glacial samples^39^. In contrast, our spICP-TOFMS measurements show augite-like particles clearly present in glacial samples (Fig. 5f), with temporal trends indicating a maximum during the LGM. This result suggests that contributions from certain sources (e.g., Patagonia) during the early Holocene was less pronounced in Taylor Glacier compared to the interior Antarctic locations such as Dome C in East Antarctica. Detection of a small number of carbonate particles during the LGM could serve as evidence of potential (although minor) input from the continental South American shelf^17^, while chlorite abundance suggests additional input from glacial deposits. This is in line with the hypothesis that dust emission from Patagonia increased when glaciers discharged directly onto outwash plains during glacial stages^18^. Clinopyroxenes (e.g. augite, pigeonite) are common in Patagonian sediments derived from erosion of widespread Andean volcanic rocks and are reported in glacial outwash deposits across Patagonia, which act as major aeolian dust emission areas^78^. However, no single mineralogical phase uniquely identifies the aeolian dust source, and augite is not exclusive to South America, as it is also reported in other sediments, including Antarctic deposits^79^ but is generally less abundant.

The tephra glass shards found in sample 14.8 kyr BP could indicate tephra deposition due to a volcanic eruption in the coastal zone of East Antarctica, which has a well-documented volcanic activity^67,80^. There are several sulphate peaks of unknown volcanic eruptions around that time (14.7, 14.9 and 15.0 kyr BP) as recorded in the composite record of average sulphate deposition in previous ice core studies^81^. There is also a tephra layer found in TALDICE (15.19 ± 0.14 kyr BP) which was attributed to explosive eruption from a Melbourne volcano^82^. Given the uncertainty in the dating of the Taylor Glacier ice core, any of the observed sulphate peaks could potentially be associated with volcanic particles present in the 14.8 kyr BP sample. However, this interpretation is complicated by the time span represented by each sample in the Taylor Glacier horizontal ice core. Alternatively, these shards may be part of the background dust, as previously observed in other Polar ice cores^83,84^. Such particles could originate from glacial or ash deposits, either locally or from distant sources. However, long-range transport is unlikely, as the large particle size supports a local origin. The sharp, angular morphology of the shards also suggests limited change. Furthermore, the close match between the SiO_2_/(Na_2_O+K_2_O) ratio of particles measured by SEM-EDS in the 14.8 kyr BP sample and that of coastal Antarctic volcanoes supports our hypothesis that one of the Victoria land volcanoes are the source of this volcanic material (Fig. 7b). In the 12.6 and 26.9 kyr BP samples, particles with compositions similar to those identified as volcanic (in both the 14.8 kyr BP sample and the 81003G Mount Erebus volcanic glass standard) were also detected, although at lower relative abundances. The similarity includes the presence of major elements within individual particles and comparable correlations between major elements. These particles may also indicate volcanic activity, for example multiple tephra layers dated around 26-27 kyr BP have been reported in the Simple Dome ice core and attributed to Mt. Berlin^69^. However, due to age uncertainties and discrete sampling of Taylor Glacier the records cannot be directly synchronised. A study of volcanic shards in Greenland concluded that individual tephra particles found in ice cores are not always associated with sulfate peaks or increased particle deposition, but also indicated that they do not represent a continuous background input; however, only a small number (< 5 per sample) of large (> 20 µm) particles were analysed^85^. Overall, 0.4–8.4% of the microparticles in the other Taylor Glacier samples also contained detectable amounts of the six major elements (Fig. S3), potentially suggesting the presence of submicron volcanic glass shards in the Antarctic atmospheric background. This hypothesis requires additional investigation which is out of the scope of this paper.

These results demonstrate the ability of spICP-TOFMS to detect tens of thousands of submicron particles compositionally distinct particles in extremely low-volume samples emphasizing the great potential of the technique for ice core studies. This capability can support targeted analysis of samples that show specific elemental signatures. In addition, composition data from spICP-TOFMS applied to different materials, such as volcanic glass, mineral standards, and dust from known source regions, could be used to develop a reference database for reliable source apportionment.

## Materials and methods

### Taylor Glacier ice core samples

Twenty-eight discrete samples used in this study were collected from an approximately 150-meter-long horizontal ice core extracted from the Taylor Glacier in East Antarctica using procedures described in^8^. The Taylor Glacier sampling site is illustrated in Fig. 1. Dating of this ice core was reported in a previous study^54^. A list of the 28 samples, their age and the linked uncertainty are provided in Table S1. Ten samples span the early Holocene period, 9 to 11.7 kyr BP; thirteen samples span the period from 12.0 to 17.1 kyr BP; five samples are older than 17.1 kyr BP with two samples representing the LGM (21.9 and 26.8 kyr BP) with the oldest sample dated at 44.4 kyr BP.

### Sample preparation and decontamination

Each Taylor Glacier ice core sample used in this study was cut into an ice stick at the National Science Foundation Ice Core Facility in Denver with a square base (3.2 cm × 3.2 cm) and a length of ~ 70 cm. About 1–2 mm of the outer portion of each ice core stick was removed and discarded in the cold room at The Ohio State University Byrd Polar and Climate Research Center (BPCRC) (Columbus, Ohio, USA) using a series of acid-cleaned stainless-steel chisels. Each chisel was cleaned using ultrapure HNO_3_ (2% v/v) (Optima, Fisher Scientific, Hampton, New Hampshire, USA) solution before use. Approximately 1-2 mm of outer layer ice was removed from all surfaces of the ice core sticks. Samples were further decontaminated using the melter system located at BPCRC^86^. Each ice stick produced a clean liquid aliquot with a volume of around 150 mL that was collected in a 250 mL acid cleaned narrow mouth LDPE bottle (Nalgene). A 5–10 mL sub-aliquot of each sample was used for spICP-TOFMS analysis. Another 20 mL sub-aliquot was collected in a Falcon vial used for Coulter Counter analysis.

### Coulter Counter analysis of Taylor Glacier ice core samples

Taylor Glacier sample suspensions were analyzed using a Multisizer 4e Coulter Counter (Beckman Coulter, Brea, California, USA) with 400 channels in the class-100 clean room at BPCRC. The Coulter Counter was set to detect particles with volume-equivalent spherical diameters from 0.6 to 18.0 µm using a 30 µm aperture. Before analysis, NaCl was added to each suspension, and the suspension (in NaCl 0.15% w/v) was rotated 3 times top-to-bottom to ensure particles were resuspended.

The data produced by the Coulter Counter instrument consists of particle number concentrations (particles mL^−1^) and particle size distributions (bins), and total mass of particles using the average of three replicate measurements for each sample. The total mass of particles and particle mass concentrations (µg kg^−1^) were determined from particle volume distributions using an assumed particle density (equal to the average crustal density) of 2.7 g/cm^3^.

### Scanning Electron Microscopy analysis

An aliquot (20 mL) of the 14.8 kyr BP Taylor Glacier ice core sample was filtered onto a 0.1 μm polycarbonate filter in the clean room at BPCRC. A  ~ 1 cm^2^ section of the filter was cut out using pre-cleaned scissors and adhered to a standard 12.7 mm aluminum stub using double-sided carbon tape. The stub was loaded into a Leica EM ACE600 for carbon coating and then analyzed on a Thermo Scientific Quattro Environmental SEM equipped with an EDAX Octane Elect super 70 mm^2^ EDS detector at the Center for Electron Microscopy and Analysis (CEMAS) at The Ohio State University. An electron acceleration voltage of 20 kV was used at a 10 mm working distance. Random particle areas were imaged at 50,000× magnification using an electron backscatter detector and a secondary electron detector.

### Single particle ICP-Time of Flight MS analyses

In spICP-TOFMS, a liquid sample is introduced to a nebulizer where it is aerosolized. Only a fraction of this aerosol (the transport efficiency) passes through the spray chamber and enters the plasma. If a droplet contains a solid particle, the liquid is desolvated in the ICP, and the particle is vaporized, atomized, and ionized. Each particle generates a discrete ion burst about 500 µs wide^87,88^. A particle is detected if it produces a signal above the IUPAC threshold^89^ relative to the quasi-continuous background, which arises from dissolved solids, small undetectable particles, polyatomic ions^51^, and overlapping particle signals. The instrument records ion signal intensity across a full mass spectrum from m/z 14 to 250. spICP-TOFMS cannot quantitatively measure H, N, O, F, Cl, or the noble gases. A reaction gas was not used. S, P, and K suffered from spectral overlaps that prevented their measurement.

The technique faces several limitations: roughly 10–30× lower sensitivity for low-mass elements when compared to quadrupole instruments^42^, element-dependent detection limits, spectral overlaps, and large data volumes (~ 132 MB per 10-minute run). Typical sample introduction systems limit particle sizes to particles smaller than about 2–5 µm, with transport efficiency decreasing for particles larger than ~ 1 µm^51,90^. Because the quasi-continuous background is highly element and sample dependent, detection thresholds vary accordingly. Major elements, other than those listed above, are accurately measurable in particles larger than 0.467 µm in this study. For particles smaller than ~ 0.467 µm in the samples measured in this study some major elements (e.g. Si, Al, Ti) may be undetectable even when minor ones are measurable, affecting the accuracy of chemical composition and mass equivalent diameter estimates. Therefore, comparisons across samples require accounting for element and sample-specific detection limits^51^ (see Table S2). The accuracy of elemental ratios in individual particles depends on measurement uncertainty, which is constrained by Poisson statistics due to the short signal duration from each particle^52^.

A TOFWERK icpTOF-R spICP-TOFMS (Thun, Switzerland) located at Carnegie Mellon University (Pittsburgh, PA, USA) was used to measure calibration solutions and suspensions. The setup included a self-aspirating PFA-50 nebulizer (Elemental Scientific Inc., Omaha, NE, USA) in a 47 mm PFA barrel spray chamber with an O-ring-free endcap, connected via a sapphire injector (1.8 mm i.d., Meinhard, Golden, CO, USA). Vials containing the aliquots were inverted three times prior to analysis. Samples were introduced by free aspiration, and uptake rates were measured gravimetrically (OHAUS Pioneer PX224/E). Instrument parameters are listed in Table 1. The CeO^+^/Ce^+^ ratio was optimized to <3% (by optimizing the nebulizer gas flow rate) while monitoring signals from a multi-element solution containing 1 µg L^−1^ Co, In, Ce, and U.

**Table 1:** Experimental parameters of TOFWERK icpTOF-R.

Parameters	Values
Plasma power (W)	1500–1550
Plasma gas(L min^−1^)	17
Auxiliary gas(L min^−1^)	1.0
Nebulizer gas flow (L min^−1^)	1.0–1.4
Sample uptake rate (µL min^−1^), freely aspirated	~ 50–70*
60 nm Au particle, transport efficiency	10–20%*
Integration time, solution (ms)	1000
Integration time, single particle (ms)	2
Notched m/z	31, 40, 80

*Varied day to day and dependent on sample uptake rate used; measured each day.

Sample volumes delivered to the ICP and particle mass equivalent diameters were calculated using the Particle Frequency transport efficiency (NP#) method^90^. Transport efficiency was experimentally determined as a function of particle size^51^. The instrument measured a full mass spectrum from m/z 14 to 250 every 30 µs; spectra acquired every 2 ms were summed prior to be stored on the hard disk. In this study, 63 isotopes (one per element) were used to determine the elemental composition and mass equivalent diameters of individual atmospheric mineral dust particles (full isotope list in Table S2).

Most of Taylor Glacier samples (19 out of 28) were measured the same or the next day after melting, 8 samples were measured on the second or third day after melting and only one sample was measured one week after melting.

The stability of the samples over time was determined for two Taylor Glacier samples aged 9.0 and 21.9-kyr BP which were analyzed daily for nine days by spICP-TOFMS beginning the day after the ice was melted. Particle number concentration in Holocene and LGM samples had similar relative standard deviations (8.0% and 7.6% of all detected particles over the 9 days, respectively) and did not reveal any temporal trends. Similarly elemental composition of particles also remains stable over 9 measurement days in both samples.

#### Calibration solutions and particle standards used in spICP-TOFMS analysis

Suspensions of 60 nm citrate-capped Au nanoparticles (Nanocomposix, San Diego, CA, USA) diluted 458,000× to produce a suspension of 100,000 particles mL^-1^ were used to determine the transport efficiency using the Particle Frequency (NP#) method [90] *.*

Multi-element calibration solutions were made from for separate multi-element standard solutions (IV-ICPMS-71: A,B,C,D) purchased from Inorganic Ventures, Christiansburg, VA, USA) containing a total of 69 elements at 10 µg mL^−1^ (Table S3) were used together with the measured transport efficiency to determine analyte sensitivities (counts fg^−1^). The four calibration solutions were combined and serially diluted in deionized water (18.2 MΩ·cm, ThermoFisher Scientific Smart2Pure Pro) to yield 1–25 ng mL^−1^ standards. For Na, Si, K, Ca, and Fe, higher concentrations were needed to achieve sufficient signal-to-noise ratios. Thus, 1000 ng mL^−1^ stock solutions were used to add these elements at 10–250 ng mL^−1^. All solutions were prepared gravimetrically (OHAUS PX224/E). The final 69 element calibration solutions were used immediately after mixing and dilution and were confirmed to remain stable over at least an hour. The signal of each of the isotopes measured was calibrated to represent the total elemental concentration (the sum of all isotopes of the element).

#### Identifying particle signals

For each sample, Tofware software (TOFWERK AG, Switzerland) determined the average quasi-continuous background for each isotope^91^. Spectral overlaps^92^ can result from plasma gas ions (e.g., ^40^Ar⁺ overlapping ^40^Ca⁺), entrained air (e.g., ^14^N₂⁺ and ^12^C^16^O⁺ overlapping ^28^Si⁺), or plasma reactions (e.g., ^38^Ar^16^O⁺ overlapping ^54^Fe⁺). Potassium isotopes (m/z 39–41) were excluded due to interference from ^40^Ar⁺ and argon hydride (^38^ArH^+^) ions^92^. Particles producing signals below detection thresholds contribute to the quasi-continuous background signal.

A signal was identified as a “particle event” if it exceeded the detection limit (L_D_) for at least one element:

1 $$\text{Detection Limit }(\mathrm{LD}) =3.29\sqrt{{\lambda }_{bkgd}} + 2.72$$

where λ_bkgd_ is the average count rate of the quasi-continuous background signal^93^. L_D_ in mass units was obtained by multiplying by the analyte mass sensitivity, yielding the smallest detectable analyte mass. Since background intensity varies by sample, an L_D_ was calculated for every element in each sample (Table S6).

Dilution may reduce quasi-continuous background and can reduce the probability of particle signal coincidence, thereby lowering L_D_ and enabling detection of smaller particles. However, comparing samples requires using a common threshold greater than the highest element L_D_ among them.

Particle number concentration was calculated as:

2 $${\#\text{particles mL}}^{-1}=\frac{\#\text{detected particle events }}{\text{uptake rate }\times \text{ \%TE }\times \text{ measurement time}}= \frac{\#\text{ detected particles}}{\text{volume of sample to ICP}}$$

The mass of each element in a particle was derived from the background-subtracted signal intensity multiplied by analyte sensitivity (counts fg^−1^).

The mass equivalent diameter corresponds to the diameter of a sphere with the same total detected mass, assuming a crustal density of 2.7 g cm^−3^ (as has been commonly done to convert particle size distributions measured by Coulter Counter to particle mass distributions):

3 $${\mathrm{Estimated}}\;{\mathrm{mass}}\;{\mathrm{equivalent}}\;{\mathrm{particle}}\;{\mathrm{diameter}} = \sqrt[3]{{\frac{{6*{\mathrm{mass}}_{{{\mathrm{particle}}}} }}{\pi \times \rho }}}$$

Corresponding masses of the most common oxides associated with major elements (SiO_2_, Na_2_O, Al_2_O_3_, TiO_2_, MgO, CaO, Fe_2_O_3_) were calculated using the molar masses of the detected elements and their oxides.

As an alternative for oxide conversion, the contribution of oxygen in crustal particles can be estimated using the average composition of the upper continental crust, where most common minerals contain ~ 47% oxygen by mass. To account for oxygen and obtain the total oxide mass, the measured elemental mass should be multiplied by a factor of 1.89 (i.e., 1/0.53). Because the diameter of a spherical particle scales with the cube root of its mass, particle sizes calculated from elemental mass can be converted to oxide-equivalent particle sizes by multiplying the diameter by a factor of 1.23. Despite differences at the individual particle level, total particle size distributions derived using the two approaches are almost identical, as positive and negative deviations largely cancel out. The resulting spICP-TOFMS PSDs are comparable with the Coulter Counter data both for Taylor Glacier samples and for SiO_2_ particle standards (Fig. S1). The transport efficiency of particles larger than about 0.6 µm measured by spICP-TOFMS (using a conventional sample introduction system) decreases as particle mass and size increases^51^. Using an uptake rate of ~ 60 µL/minute, 700 nm engineered SiO_2_ particles have a transport efficiency of around 13%. At 0.9 µm, the transport efficiency decreases to about 9%, while particles ≥ 1.0 µm have low transport efficiencies (< 5%)^51^. Therefore, only particles with diameters greater than 0.6 µm and less than 1.0 µm were considered when comparing measurements made by the two techniques. Larger discrepancies are observed for low-concentration Holocene samples, likely reflecting increased uncertainty in Coulter Counter measurements. On average, total particle number and mass concentrations measured by the Coulter Counter are ~ 1.5–2 times higher than those measured by spICP-TOFMS for high-concentration samples and standards (Fig. S1b). This difference is likely due to the reduced transport efficiency of larger particles in spICP-TOFMS and underestimation of particle mass for particles containing elemental components that are not detected by spICP-TOFMS.

The smallest particle mass-equivalent diameter at which the total particle number concentration can be accurately determined can be estimated by fitting a Pareto distribution to the upper end of the measured mass-equivalent diameter distribution. At this upper end, masses of most major detectable elements, such as Si, Al, and Fe in particles in the ice core samples, are greater than the highest limit of detection.

Detection limits are element- and sample-dependent. Elements that are less abundant but have low background levels can often be detected in particles smaller than 0.467 µm, whereas major elements may not be detectable. Since our approach focuses on the major elemental composition of the particles, trace and rare earth elements are not discussed.

The deviation between the particle number concentration predicted by the Pareto function and the measured particle number concentration as a function of particle mass-equivalent diameter can be used to roughly estimate the smallest mass-equivalent diameter and therefore the smallest total particle mass below which the number concentration of particles is underestimated. Using a Pareto fit increases the likelihood that only particles containing a detectable amount of Si, a common constituent of crustal particles with a high quasi-continuous background, are compared.

This approach also defines a threshold for comparing particle number concentrations among samples. The mass-equivalent diameter at which the Pareto fit deviates from the measured particle distribution is used as this threshold. Only particles larger than the largest threshold among all samples can be fairly compared. Particles smaller than this threshold can still provide information on the mass of detectable elements but not on particle mass-equivalent diameter or the mass fraction of elements relative to total particle mass. This limitation also applies to nanoparticles, which may contain measurable elements without allowing determination of complete elemental composition or accurate mass-equivalent diameter if other elements are below the detection limit.

Specific examples from Taylor Glacier illustrate this approach (Fig. S7). In the 12.6 kyr BP sample, the Pareto deviation occurs at a mass-equivalent diameter smaller than 237 nm. In the LGP sample of 32.2 kyr BP, the deviation occurs at a mass-equivalent diameter smaller than 0.467 µm. Although particles smaller than 467 nm are present in the LGP sample, their mass-equivalent diameters cannot be accurately calculated due to high Si and Al quasi-continuous backgrounds. Only the number concentration of particles larger than 467 nm can be fairly compared among these samples. The decline in both Si- and Al-containing particle counts toward smaller diameters reflects a particle-level detection limit rather than elemental absence (Fig. S7e). In mineral dust, Al is predominantly present in aluminosilicate phases together with Si, and its mass therefore decreases in proportion to particle volume. As particle size approaches the detection limit, the total elemental mass becomes insufficient to trigger reliable particle identification, leading to an undercounting of both Si- and Al-bearing particles. At smaller diameters, however, the relative abundance of Al-containing particles increases again until the particles are too small to detect Al. This reflects the emergence of a distinct population of Al-rich (e.g. Al enrich clay minerals), particles that remain detectable. Similarly, the increase in total number of particles in even smaller sizes reflects the Ti-rich particles. Therefore, the observed particles size distribution at diameters smaller than 0.467 µm represents a detection artifact.

The effect of sample dilution on the quasi-continuous background was also assessed for the 21.9 kyr and 26.8 kyr samples (these had the highest particle concentrations and quasi-continuous backgrounds). Diluting these samples with deionized water reduced the background signal and lowered the measured mass-equivalent diameter at which the Pareto fit deviates. The 21.9 and 26.8 kyr BP Taylor Glacier ice core samples originally had high Pareto thresholds of 0.875 and 0.977 µm, respectively, due to elevated concentrations of mineral dust aerosol particles and high Si and Al quasi-continuum background signals. Dilution by approximately tenfold with ultrapure deionized water reduced the Pareto thresholds to 0.442 µm for the 21.9 kyr BP sample and 0.404 µm for the 26.8 kyr BP sample (Fig. S7). Further dilution did not improve the values of L_D_ and minimum accurately measurable particle size because background Si concentrations approached those in deionized water. No samples had a Si-mass threshold low enough to determine the mass-equivalent diameters of particles smaller than 0.1 µm.

The largest particles measured by spICP-TOFMS in each sample have mass-equivalent diameters of approximately 2.5 µm or less. Although larger “fine microparticles” are present (detected by Coulter Counter as shown in Fig. S1d–i), mass- and size-dependent transport efficiency limits the number of these particles delivered to the ICP^50^. Although particles smaller than 1 µm account for less than approximately 10 percent of the total particle mass measured by Coulter Counter, they represent the majority of particles by number.

Considering particles larger than 0.467 nm but smaller than approximately 2.5 µm ensures that results obtained using spICP-TOFMS are representative and statistically significant. It should be noted that comparing these results to other techniques, such as bulk ICP-SFMS, requires caution, as several large particles digested in acid could disproportionately influence total element concentrations and obscure potential source fingerprints in the majority of particles.

#### spICP-TOFMS data processing

Analysis of spICP-TOFMS data was performed using a Python based tool produced in our lab to process the output files from the TOFWERK software to calculate transport efficiency and the calibrated mass of each element within individual particle. The calculations were additionally verified using an Excel template spreadsheet and produced identical results. The spICP-TOFMS does not measure oxygen, which is a major constituent of most particles composed of oxides. To account for that and to make it comparable with measurements using different analytical techniques we converted elemental concentrations into oxide concentrations^62^.

#### Artificial ice samples to assess potential contamination from the decontamination and melting process

Laboratory-made ice was used to test the cleanliness of ice core melting and processing procedure, in terms of water-insoluble contaminant particles. An artificial ice core (half-cross section) was made by freezing ultrapure 18.2 MΩ-cm deionized water in an acid cleaned 3 L perfluoroalkoxy alkane (PFA) container (Savillex). The artificial ice was then removed from the container, cut into rectangular sticks on a band saw in the cold room at -5 °C at BPCRC, bagged in plastic lay flat bags and processed the same way as the Taylor Glacier ice samples. Melted artificial ice measured typically contained <200 particles mL^-1^ with particles >0.467 µm virtually absent (as measured by spICP-TOFMS, as described below).

#### Measurements of the volcanic glass standard

A secondary glass standard, 81003G from Erebus Volcano (matrix glass separated from lava bombs erupted from Erebus volcano in Nov 1981) which is widely used as an internal standard for geochemical analysis^67^ was measured using spICP-TOFMS. These samples were ground down to a coarse mineral powder by using a mortar and pestle. Approximately 1 g of the resulting tephra powder was suspended in 1 mL of deionized water. The resulting concentrated suspension was diluted by 20,000 and measured by spICP-TOFMS. 60,459 particles were detected in total in 10 min.

We compared bulk major oxide concentrations calculated from spICP-TOFMS measurements and measured by XRF^67^ for major oxides. Most oxides (SiO_2_, Al_2_O_3_, Na_2_O, FeO, TiO_2_, MgO, MnO) fall close to the 1:1 line, indicating strong agreement between the two methods (Fig. S8). Concentration (wt%) of CaO measured by spICP-TOFMS is 0.6 wt% which is significantly lower than 2 wt% measured by XRF suggesting an underestimation of CaO in particles by the spICP-TOFMS. The detection of low amounts of Ca in particles is challenging due to high background signals across all Ca isotopes. Even the most favorable isotope for detection, m/z 44, is affected by dissolved Ca in the samples and by spectral interferences from overlapping ions (e.g., ^12^C^16^O_2_^+^). A comparison between trace element concentrations measured by spICP-TOFMS and LA-ICP-MS reveals a consistency between two methods for most of the measured elements and elemental patterns. Larger discrepancies were found for heavy rare earth elements (e.g., Eu-Lu) which were significantly underestimated by the spICP-TOFMS likely due to insufficient number of particles detected containing these elements. E.g. there were only 15 particles out of 60,459 detected, containing Eu and 5 particles containing Lu. This is also confirmed by the better agreement of quasi-continuous background values of heavy rare earth elements measured by spICP-TOFMS with LA-ICP-MS data as it includes both dissolved ions in the carrier solution as well as discrete particles with masses of elements below the detection limits for individual particles (Fig. S8).

#### Comparison of spICP-TOFMS and bulk analysis of acidified Taylor Glacier samples

A first approximation of the “bulk” concentration of elements measured by spICP-TOFMS (referred to as “bulk spICP-TOFMS”) can be determined by summing total mass of particles (signals above the element threshold) divided by the volume of sample that entered the plasma and the total mass concentration calculated from the quasi-continuous background (signal below the element threshold). However, the mass of elements in particles only includes particles smaller than ~ 2.5 µm. Bulk spICP-TOFMS concentrations in particles (in ng mL^−1^) were always less than that of the concentration calculated from the quasi-continuous background signal. The element concentrations measured by spICP-TOFMS from the quasi-continuous background signal exist in the sample as dissolved ions in the carrier solution as well as discrete particles with masses of elements below the detection limits for individual particles, leading to a higher total mass in the background. This results in some of the particle mass being measured as part of the background rather than as distinct particle events. Signals due to spectral overlaps would also contribute to the quasi-continuous background but not to the particle signals on top of the quasi-continuous background.

The bulk elemental composition of the Taylor Glacier ice core was previously measured using Inductively Coupled Plasma Sector Field Mass Spectrometry (ICP-SFMS) of acidified samples^8^. However, a direct comparison between ICP-SFMS and spICP-TOFMS elemental data is difficult. The samples measured using ICP-SFMS were acidified for approximately 1 month using 2% HNO₃ to dissolve the insoluble fraction, including large particles (particles too large to be efficiently transported from the sample, through the spray chamber and into the ICP. Thus ICP-SFMS measurements cannot distinguish between dissolved insoluble particles, soluble particles, and free ions of the same element in the sample. In contrast, spICP-TOFMS analyzes only particles within a limited but well-defined size range (0.467–2.5 µm). Particles > 2.5 µm cannot be analyzed which leads to an underestimation of the total mass of elements within particles in the sample. Our data of particles elemental composition are representative of the atmospheric background MPs without the influence of large (> 2.5 µm) and very large (> 5 µm) particles which were found to significantly contribute to the total mass and bulk composition of the samples during the Holocene^8,19^.

Absolute concentrations (mass/liquid sample volume) due to elements in submicron particles measured by spICP-TOFMS were, on average, about an order of magnitude lower than those in the quasi-continuous background and bulk ICP-SF-MS, consistent with our observation that submicron particles account for ~ 10% of the total particulate mass. Bulk concentrations determined by ICP-SFMS on acidified Taylor Glacier samples are in close agreement with quasi-continuous background values measured by spICP-TOFMS (Table S4). The elemental ratios of LGM to Holocene concentrations varies across methods but shows consistent overall variation patterns (Table S4). Particle-resolved measurements by spICP-TOFMS indicate higher LGM/Holocene ratios for most major elements, with particularly large differences for Na (150), Mg (97), and Mn (116). Quasi-continuous background measurements performed by spICP-TOFMS, which include dissolved species, particles below the detection limit and (for some elements/isotopes) spectral overlap ions, yield lower ratios (2–15) for many elements, although Al, Ti, Mn, and Ba still showed marked contrasts between periods. Na measured in particles by spICP-TOFMS exhibits a much larger LGM/Holocene ratio (150) compared to both quasi-continuous background (6) and bulk ICP-SF-MS results (5), likely reflecting a dominant but less variable contribution of soluble Na, across different climatic periods. The lower ratio obtained for Fe in the quasi-continuous background, together with the higher Holocene concentration (~ 7 ng mL^−1^), may reflect spectral overlaps at mass-to-charge (m/z) 54 (e.g., ^40^Ar^14^N^+^; ^38^Ar^16^O^+^; ^36^Ar^18^O^+^). Such interferences do not affect particle measurements (other than their detection limits) but can influence quasi-continuous background determinations and detection limits of elements in particles, especially for low-concentration samples. Despite the methodological differences, all approaches consistently show substantially higher elemental concentrations during the LGM compared to the Holocene.

### Mineral classification approach using normalized elemental ratios

Individual particles measured by spICP-TOFMS were classified into mineralogical categories based on their measured elemental mass fractions (Table S5). The classification of single particles using elemental composition is a well-established approach in SEM-EDS studies^94^. More recently, machine learning classifiers have been developed to rapidly assign mineral identity based on elemental mass fraction measurements of particulate mineral standards^95^. In this work, minerals were identified using characteristic elemental combinations and molar ratios, primarily normalized to Si, as summarized in Table S5.

To calculate molar fractions, elemental masses were first divided by their respective average atomic masses and then normalized to the total molar sum in each particle. Pyroxenes, such as augite-like and diopside-like particles, were defined using Ca, Mg, and Fe thresholds and specific ratios relative to Si. Amphiboles were distinguished by high Fe or Mg content in combination with Si, Al, and Ti. Micas and clay minerals were classified based on Al, Mg, Fe, and Ca content, with criteria reflecting typical mineral formulae. Feldspar-like particles, including albite-like and anorthite-like types, were assigned using Na, Ca, and Al ratios to Si. Quartz-like particles were defined by a high Si fraction greater than 0.9. Particles that were dominated by a single element, such as Fe-rich particles resembling hematite or goethite, were assigned to separate categories.

Each classification rule incorporates an arbitrary tolerance range to account for natural mineral variability, analytical uncertainty, and the lack of structural or phase information. For example, chlorite particles can have an (Fe+Mg+Al)/Si ratio of approximately 2 to 3, but a broader range of 0.95 to 3.6 was applied to capture the full diversity of particle compositions. Because the classification includes only a limited set of elements, with O, H, and K not measured, overlapping assignments are possible. Consequently, some particles were classified into multiple categories, such as biotite and phyllosilicate-like, or augite and diopside-like, which means that the total percentage of identified minerals in a sample may exceed 100 percent.

## Supplementary Information

Below is the link to the electronic supplementary material.

Supplementary Material 1

## Data Availability

spICP-TOFMS data are available at https://www.ncei.noaa.gov/access/paleo-search/study/40380.
