# Supplementary material for: Geochemical characterization of millions of individual atmospheric particles entrapped in Antarctic ice across the last glacial-interglacial transition
Source: Sci Rep. 2026 Mar 30;16:10556. doi: 10.1038/s41598-026-45260-3 (PMC13040075; doi:10.1038/s41598-026-45260-3)
Supplement: Supplementary file 1 — Supplementary Material 1 [file 41598_2026_45260_MOESM1_ESM.docx]

Supplementary Materials for

**Geochemical Characterization of Millions of Individual Atmospheric Particles Entrapped in Antarctic Ice across the Last Glacial-Interglacial Transition**

Stanislav Kutuzov *et al.*

*Corresponding author. Email: kutuzov.1@osu.edu

**This file includes:**

Fig. S1 to S8

Tables S1-S6

Figure S1.

**a,** Number size distributions of mineral particles measured by spICP-TOFMS for 9.2, 14.8 and 21.9 kyr BP Taylor Glacier samples, shown as a function of mass-equivalent diameter; dashed lines indicate power-law fits with corresponding slopes (*b*). Number size distributions for both techniques are presented using linear diameter binning**. b,** Comparison of total particle number concentrations measured by spICP-TOFMS and Coulter Counter 0.6-1.0 µm size range) on a log-log scale. **c,** Example number size distributions measured by Coulter Counter (black) and spICP-TOFMS (red) for SiO_2_ particle standards (Bangs Laboratories) with nominal diameters of 690 and 895 nm. spICP-TOFMS sizes are reported as SiO_2_-equivalent diameters assuming spherical particles and a density of 2.65 g cm^-3^. **d–f,** Number size distributions for for 9.2, 14.8 and 21.9 kyr BP Taylor Glacier samples measured by Coulter Counter (black) and spICP-TOFMS (red), highlighting the overlapping size range and divergence at larger diameters. The 21.9 kyr BP sample (i) was analysed after tenfold dilution, and spICP-TOFMS data are shown after correction by the dilution factor. **g–i,** Corresponding particle mass concentration distributions as a function of mass-equivalent diameter. **j–l,** Enlarged views of the 0.6>1.5 µm particle diameter range. spICP-TOFMS sizes are reported as major element oxides equivalent diameters assuming spherical particles and a density of 2.7 g cm^-3^.


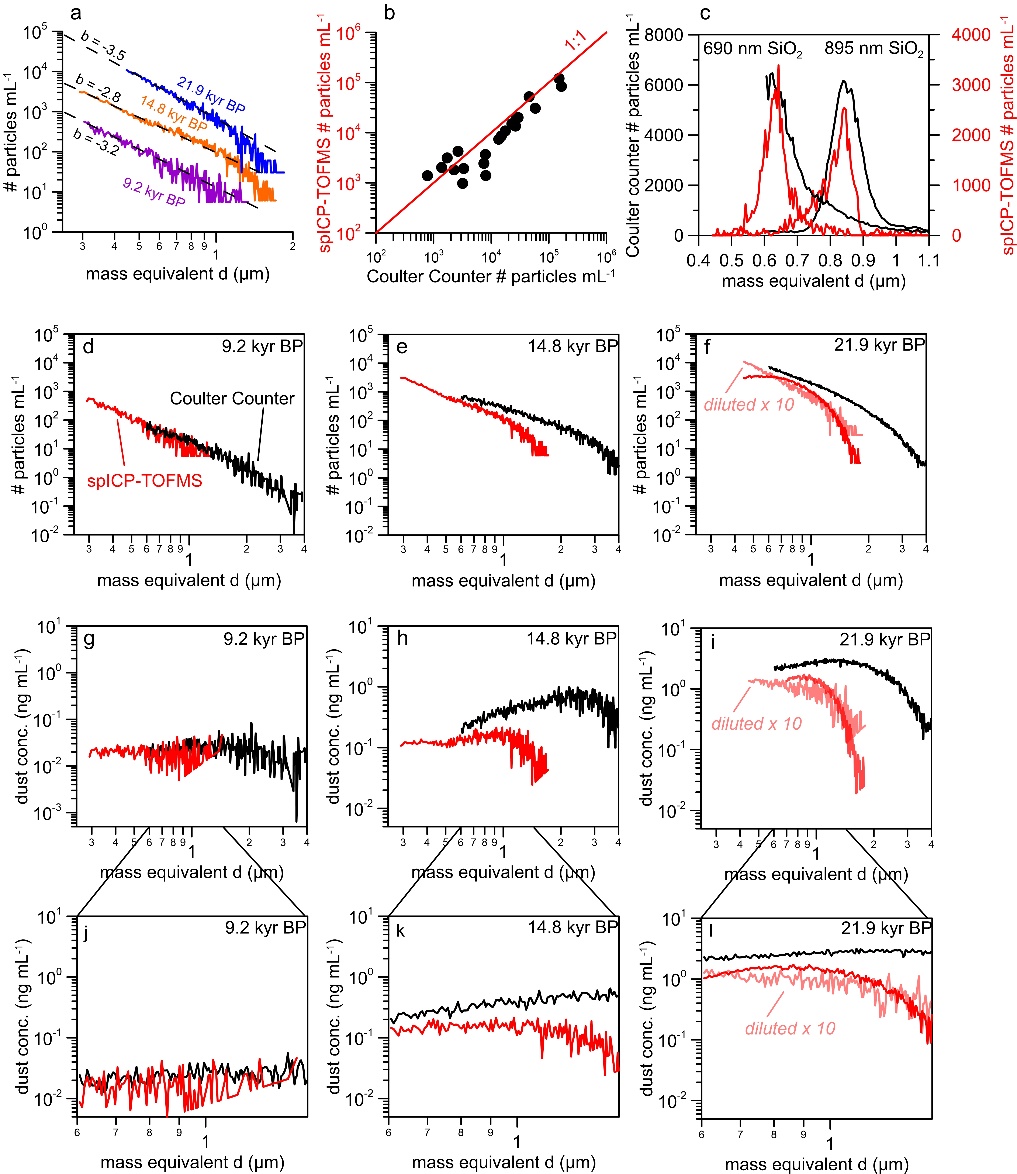


Figure S2.

Major elements (sums of all masses of each element in particles (d>0.467 µm)) expressed as oxides in particles detected by spICP-TOFMS in Taylor Glacier samples. UCC reference is shown for comparison [59]. Ages of the samples measured in this study are shown by black dots.


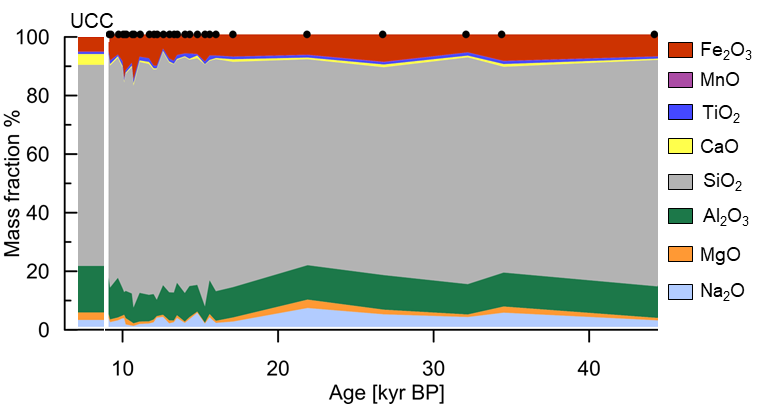


Figure S3.

Age dependent percentages of microparticles larger than 0.467 nm containing detectable amounts of: **a** Na, **b** Mn, **c** both Mn and Sr, **d** of six major elements (Na, Al, Si, Fe, Mn, Ti), and **e** same 6 major elements as in d plus Ca in Taylor Glacier samples as well as in 81003G volcanic glass standard. Note sample 14.8 kyr BP which has a distinct elemental signature suggesting volcanic particles input.


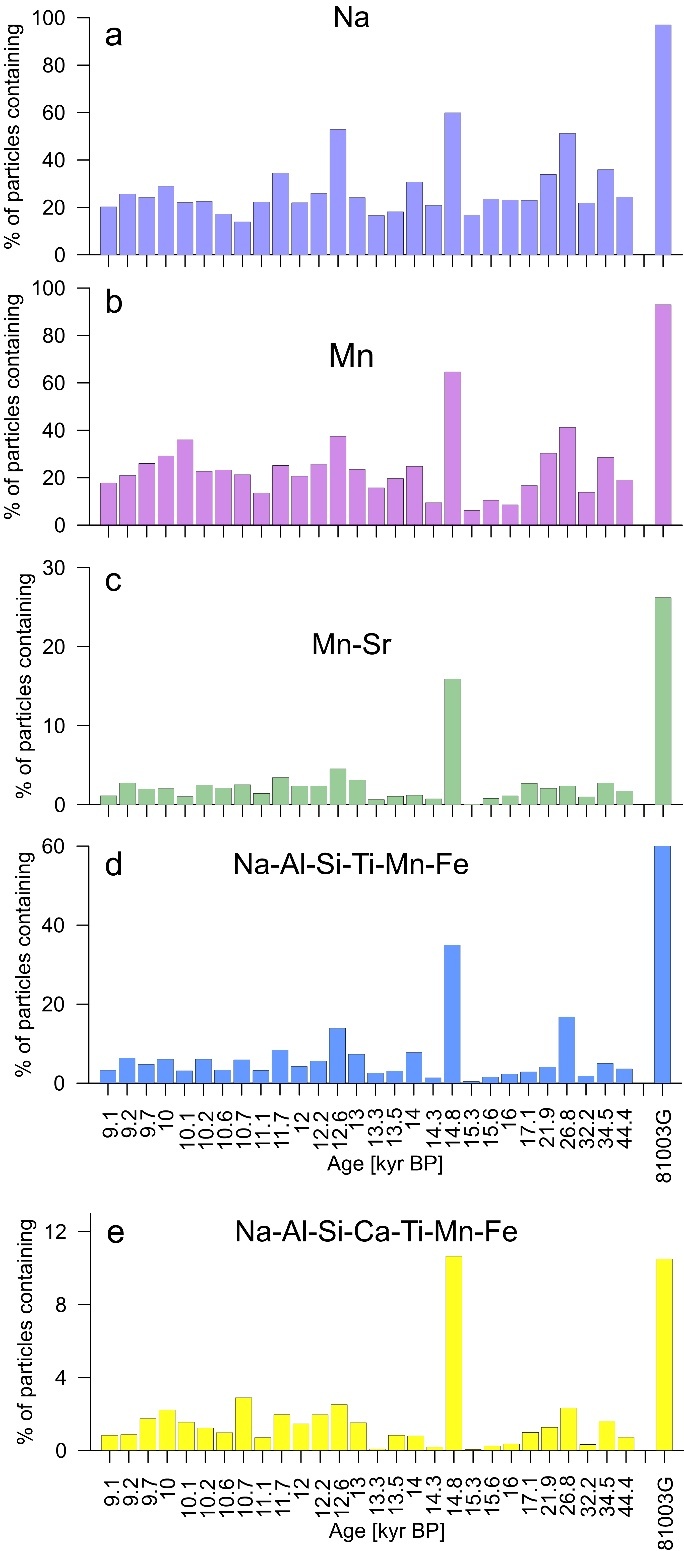


Figure S4.

Boxplots of normalized oxide mass fractions (wt%) for selected major oxides in individual particles measured in Taylor Glacier samples. **a** Na_2_O, **b** MgO, **c** Al_2_O_3_, **d** SiO_2_, **e** Fe_2_O_3_, **f** CaO. Each box shows the interquartile range (IQR), with medians as horizontal lines and whiskers extending to 1.5*IQR. Includes only particles larger than 467 nm that contain detectable amounts of the element listed in each plot.


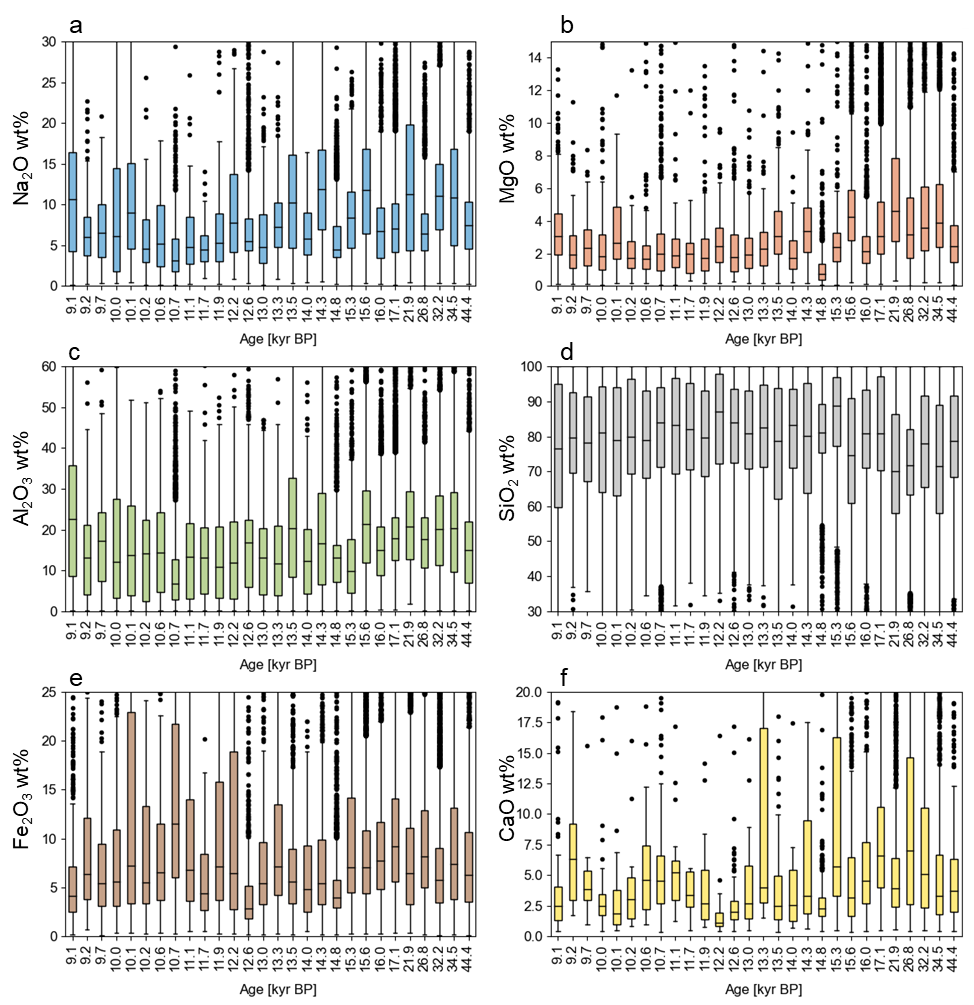


Figure S5.

Ternary diagrams showing the relative oxide compositions (Fe_2_O_3_-MgO-CaO) of individual particles analyzed by spICP-TOFMS in (**a**) Holocene (9.1-11.7 kyr BP) and (**b**) Glacial (21.8-34.5 kyr BP) samples. Each point represents a single particle larger than 467 nm, plotted according to its Fe_2_O_3_, MgO, and CaO fractions.


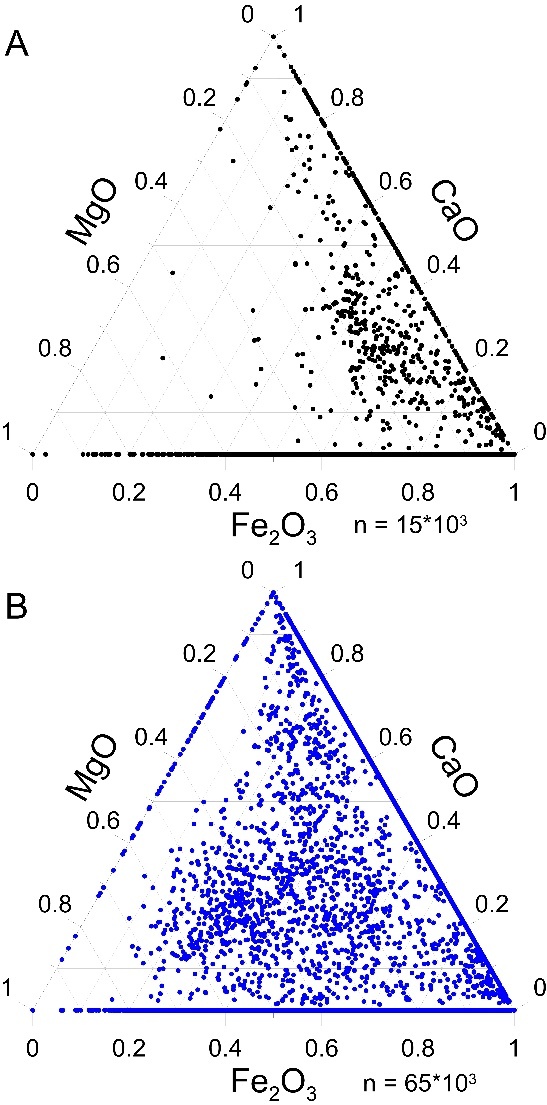


Figure S6.

Correlations of elements in individual particles d>0.467 µm. (**a-p)** Scatter plots show relationships between elements in fg measured by spICP-TOFMS. Only particles with detected major elements (Na, Al, Si, Ti, Fe, Mn) are considered. Blue symbols represent particles from the 12.6 kyr BP sample, orange symbols from the 26.9 kyr BP sample, red symbols from the 14.8 kyr BP sample, and black symbols correspond to measurements of the 81003G Mount Erebus volcanic glass. Subplots are arranged as paired comparisons (a-b, c-d, e-f, g-h, i-j, k-l, m-n, o-p), where each pair displays the same elemental relationship for 12.6 and 26.9 kyr BP samples (left panels) and for comparison with the 14.8 kyr BP sample and 81003G Mount Erebus volcanic glass reference material (right panels).


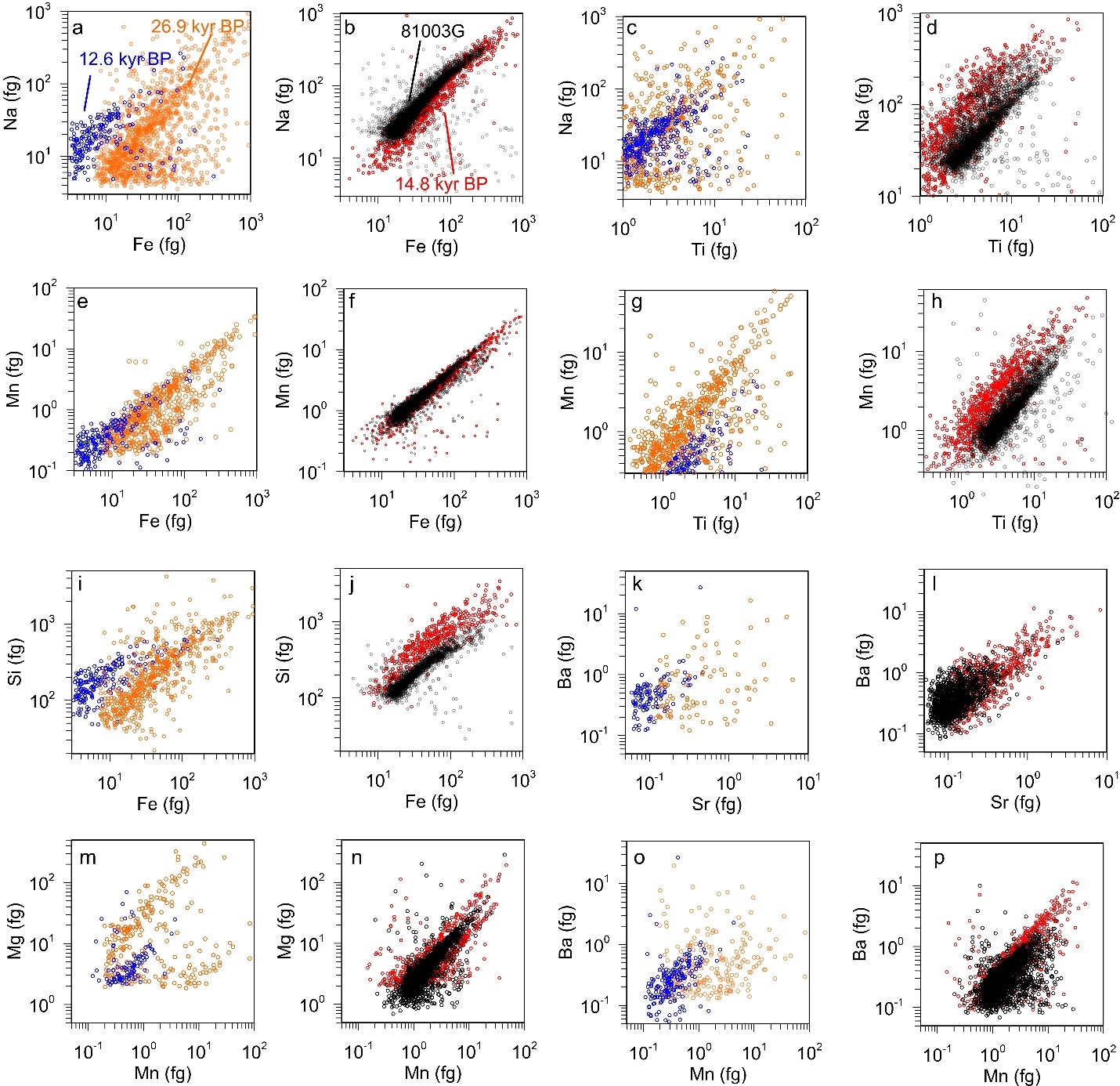


Figure S7.

Mass equivalent diameter distributions of (**a**) 12.6 kyr BP, (**b**) 32.2 kyr BP, (**c**) original 21.9 kyr BP and (**d**) 10x diluted 21.9 kyr BP Taylor Glacier samples. A Pareto distribution (dashed line) indicating the smallest accurately calculable mass equivalent diameter (red line) was fit to each sample. Number concentration vs. mass equivalent diameter of a 32.2 kyr BP Taylor Glacier sample (**e**). The mass equivalent diameter distributions of Si-containing (orange), Al-containing (green), and all particles (black) are shown. Al and Si thresholds (expressed as a mass equivalent diameter of a particle containing only that element with a density of 2.7 g/cm^3^) are shown (green and orange dashed lines).


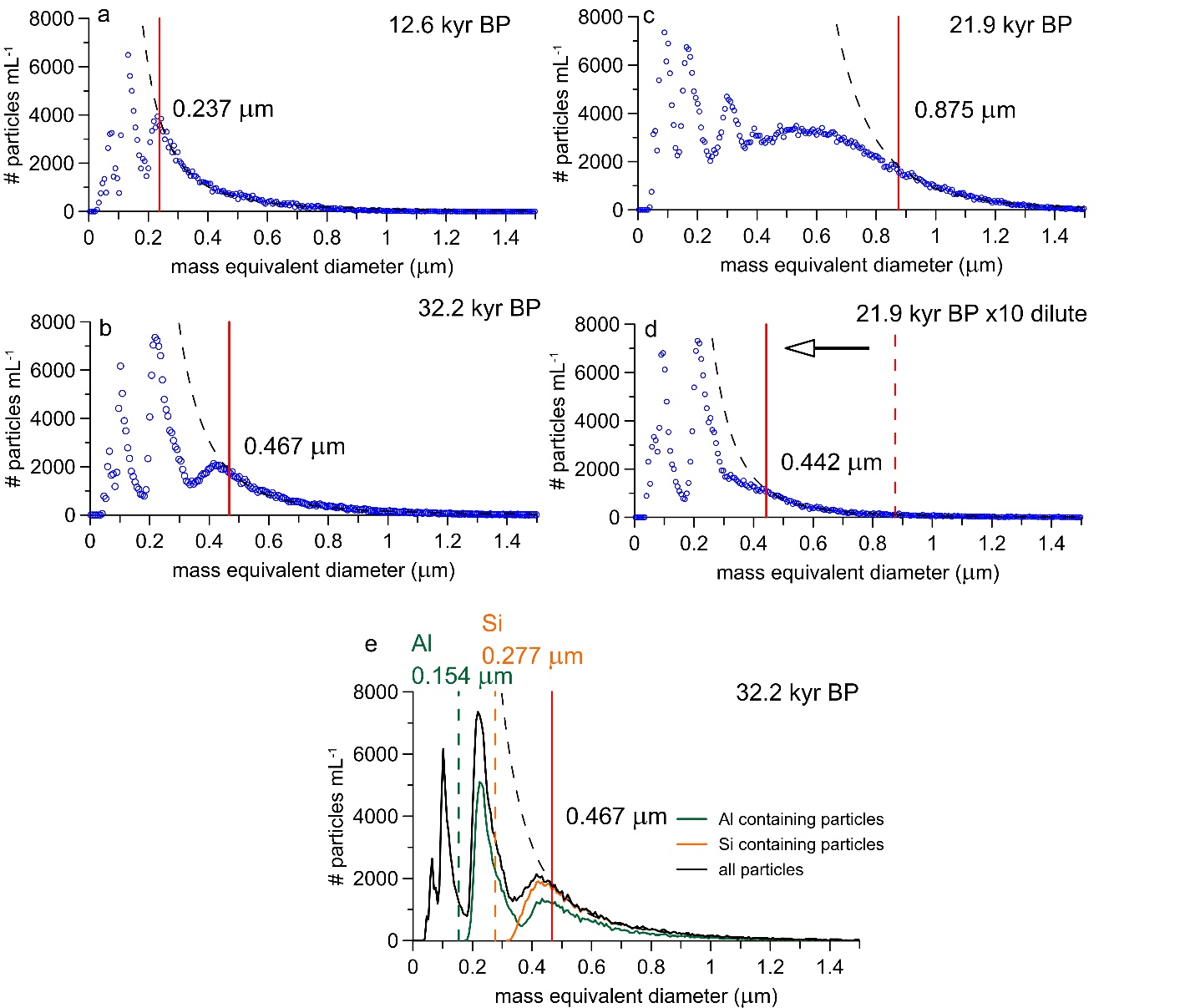


Figure S8.

Comparison of elemental compositions of 81003G Erebus glass standard measured using “bulk” spICP-TOFMS, XRF [67] and LA-ICP-MS [67]. (**a**) Major oxide wt% measured by spICP-TOFMS and XRF. (**b**) Trace element concentrations measured by spICP-TOFMS compared to LA-ICP-MS. (**c**) Trace element patterns measured by spICP-TOFMS and LA-ICP-MS.


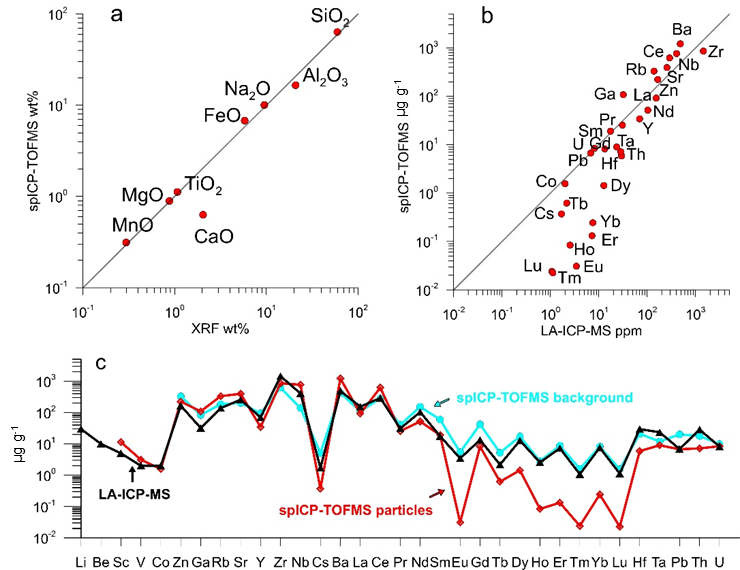


Table S1.

Taylor Glacier samples, age and age uncertainties, sample volumes, particles detected, and particle number concentrations measured by spICP-TOFMS

| Sample  name | Sample age  (kyr BP) | spICP-TOFMS | | | |
| --- | --- | --- | --- | --- | --- |
|  |  | Volume  to plasma (mL) | # particles  detected, (thousands) | # particles mL^-1^ > 0.467 µm threshold (thousands) | Conc. (ng mL^-1^)  > 0.467 µm threshold |
| 184TG-P205 | 9.1±0.2 | 0.30 | 37.1 | 7.4 | 5.0 |
| 184TG-P200 | 9.2±0.1 | 0.18 | 15.9 | 4.4 | 2.5 |
| 184TG-P180 | 9.7±0.1 | 0.17 | 9.4 | 2.3 | 1.6 |
| 184TG-P170 | 10.0±0.2 | 0.12 | 23.7 | 9.3 | 5.6 |
| 184TG-P165 | 10.1±0.1 | 0.33 | 10.1 | 1.2 | 0.6 |
| 184TG-P160 | 10.2±0.1 | 0.16 | 19.8 | 3.9 | 2.6 |
| 184TG-P145 | 10.6±0.1 | 0.17 | 14.8 | 4.8 | 5.6 |
| 184TG-P140 | 10.7±0.1 | 0.45 | 36.8 | 17.7 | 2.7 |
| 184TG-P125 | 11.1±0.1 | 0.18 | 12.1 | 3.1 | 1.8 |
| 184TG-P105 | 11.7±0.1 | 0.17 | 6.7 | 1.2 | 0.8 |
| 184TG-P100 | 12.0±0.2 | 0.17 | 13.3 | 4.4 | 3.0 |
| 184TG-P95 | 12.2±0.4 | 0.33 | 11.0 | 2.2 | 1.2 |
| 184TG-P85 | 12.6±0.2 | 0.12 | 22.7 | 25.2 | 13.8 |
| 184TG-P80 | 13.0±0.05 | 0.16 | 20.4 | 7.5 | 4.7 |
| 184TG-P70 | 13.3±0.2 | 0.18 | 17.2 | 6.1 | 3.1 |
| 184TG-P65 | 13.5±0.2 | 0.22 | 23.8 | 11.8 | 5.1 |
| 184TG-P50 | 14.0±0.1 | 0.17 | 12.5 | 2.9 | 1.7 |
| 184TG-P45 | 14.3±0.1 | 0.56 | 57.9 | 5.6 | 2.0 |
| 184TG-P35 | 14.8±0.1 | 0.16 | 33.0 | 33.3 | 27.0 |
| 184TG-N91 | 15.3±0.1 | 0.18 | 30.4 | 22.2 | 13.8 |
| 184TG-P25 | 15.6±0.3 | 0.45 | 124.9 | 49.9 | 23.3 |
| 184TG-P20 | 16.0±0.1 | 0.18 | 47.6 | 63.1 | 37.6 |
| 184TG-N105 | 17.1±0.2 | 0.45 | 82.9 | 91.0 | 85.8 |
| 184TG-N119 | 21.9±0.8 | 0.33 | 822.8 | 288.5 | 207.3 |
| 184TG-N133 | 26.8±0.8 | 0.18 | 439.4 | 417.3 | 279.8 |
| 184TG-N154 | 32.2±1.0 | 0.35 | 69.0 | 61.6 | 38.6 |
| 184TG-N161 | 34.5±0.5 | 0.12 | 43.5 | 153.5 | 101.2 |
| 184TG-N196 | 44.4±0.5 | 0.17 | 84.7 | 44.4 | 29.6 |

*Age dates and uncertainties provided by Baggenstos et al., 2018

Table S2.

Elements (isotopes) measured during spICP-TOFMS analysis of Taylor Glacier ice core samples. Bolded elements (isotopes) were used to determine the total sum of detectable elements in each particle and calculate particle mass equivalent diameter. The calibration of signal intensity versus mass for each isotope was based on the total mass of all isotopes for that element.

| **^23^Na** | ^61^Ni | **^93^Nb** | ^123^Sb | ^156^Gd | ^186^W |
| --- | --- | --- | --- | --- | --- |
| **^24^Mg** | ^62^Ni | ^94^Zr | ^124^Sn | **^157^Gd** | **^187^Re** |
| ^25^Mg | **^63^Cu** | **^95^Mo** | ^125^Te | ^158^Gd | ^188^Os |
| ^26^Mg | **^64^Zn** | ^96^Mo | ^126^Te | **^159^Tb** | ^189^Os |
| **^27^Al** | ^65^Cu | ^97^Mo | ^128^Te | ^160^Gd | **^190^Os** |
| **^28^Si** | ^66^Zn | ^98^Mo | **^130^Te** | ^161^Dy | ^191^Ir |
| ^29^Si | ^67^Zn | ^99^Ru | ^132^Ba | ^162^Dy | ^192^Os |
| ^30^Si | ^68^Zn | ^100^Ru | **^133^Cs** | **^163^Dy** | **^193^Ir** |
| ^39^K | **^69^Ga** | **^101^Ru** | ^134^Ba | ^164^Dy | ^194^Pt |
| ^40^Ca | ^70^Ge | ^102^Ru | ^135^Ba | **^165^Ho** | **^195^Pt** |
| ^41^K | ^71^Ga | **^103^Rh** | ^136^Ba | ^166^Er | ^196^Pt |
| ^42^Ca | **^72^Ge** | ^104^Ru | ^137^Ba | **^167^Er** | **^197^Au** |
| ^43^Ca | ^73^Ge | **^105^Pd** | **^138^Ba** | ^168^Er | ^198^Pt |
| **^44^Ca** | ^74^Ge | ^106^Pd | **^139^La** | **^169^Tm** | ^199^Hg |
| **^45^Sc** | **^75^As** | **^107^Ag** | **^140^Ce** | ^170^Er | ^200^Hg |
| ^46^Ti | ^76^Se | ^108^Pd | **^141^Pr** | ^171^Yb | ^201^Hg |
| ^47^Ti | ^77^Se | ^109^Ag | ^142^Ce | **^172^Yb** | ^202^Hg |
| **^48^Ti** | **^78^Se** | ^110^Cd | **^143^Nd** | ^173^Yb | ^203^Tl |
| ^49^Ti | ^79^Br | **^111^Cd** | ^144^Nd | ^174^Yb | ^204^Pb |
| ^50^Ti | ^80^Se | ^112^Cd | ^145^Nd | **^175^Lu** | **^205^Tl** |
| **^51^V** | ^81^Br | ^113^Cd | ^146^Nd | ^176^Yb | ^206^Pb |
| **^52^Cr** | ^82^Se | ^114^Cd | **^147^Sm** | ^177^Hf | ^207^Pb |
| ^53^Cr | **^85^Rb** | **^115^In** | ^148^Sm | **^178^Hf** | **^208^Pb** |
| **^54^Fe** | ^86^Sr | ^116^Sn | ^149^Sm | ^179^Hf | **^209^Bi** |
| **^55^Mn** | ^87^Rb | ^117^Sn | ^150^Sm | ^180^Hf | **^232^Th** |
| ^56^Fe | **^88^Sr** | ^118^Sn | ^151^Eu | **^181^Ta** | ^234^U |
| ^57^Fe | **^89^Y** | ^119^Sn | ^152^Sm | ^182^W | ^235^U |
| **^58^Ni** | **^90^Zr** | **^120^Sn** | **^153^Eu** | ^183^W | **^238^U** |
| **^59^Co** | ^91^Zr | **^121^Sb** | ^154^Sm | **^184^W** |  |
| ^60^Ni | ^92^Zr | ^122^Sn | ^155^Gd | ^185^Re |  |

Table S3.

Multi-element solutions from Inorganic Ventures used to make calibration solutions.

| Acid matrix | Solution name | Elements |
| --- | --- | --- |
| 3% v/v HNO_3_ | Solution A | Al, As, B, Ba, Be, Cd, Ca, Ce, Cr, Co, Cs, Cu, Dy, Er, Eu, Gd, Ga, Ho, Fe, La, Pb, Lu, Mg, Mn, Nd, Ni, P, K, Pr, Rb, Sm, Se, Ag, Na, Sr, S, Tl, Th, Tm, U, V, Yb, Zn |
| 2% v/v HNO_3_ +  trace HF | Solution B | Ge, Hf, Mo, Nb, Sb, Si, Sn, Ta, Te, Ti, W, Zr |
| 30% v/v HCl | Solution C | Au, Ir, Os, Pd, Pt, Re, Rh, Ru |
| 3% v/v HNO_3_ | Solution D | Bi, In, Li, Sc, Tb, Y |

Table S4.

Selected LGM/Holocene concentration ratios and concentrations determined in the Taylor glacier ice core.

| **LGM/Holocene ratio** | Na | Mg | Al | Si | Ca | Ti | Fe | Mn | Ba |
| --- | --- | --- | --- | --- | --- | --- | --- | --- | --- |
| spICP-TOFMS due to particles | 150 | 97 | 71 | 48 | 70 | 38 | 37 | 116 | 62 |
| spICP-TOFMS quasi-continuous background | 6 | 15 | 68 | 2 | 4 | 52 | 6 | 29 | 30 |
| ICP-SFMS [8] | 5 | - | 46 | - | - | - | 38 | 50 | 34 |
| **Concentrations** |  | | | | | | | | |
| spICP-TOFMS Concentration due to particles (Holocene) (ppb) | 0.06 | 0.02 | 0.15 | 1.1 | 0.01 | 0.02 | 0.2 | 0.001 | 0.001 |
| spICP-TOFMS Concentration due to particles (LGM) (ppb) | 8.4 | 1.9 | 10.3 | 53.6 | 0.001 | 0.86 | 8.4 | 0.17 | 0.05 |
| spICP-TOFMS quasi-continuous background (Holocene) (ppb) | 29.1 | 3.2 | 1.2 | 197.2 | 16.4 | 0.06 | 7.2 | 0.05 | 0.02 |
| spICP-TOFMS quasi-continuous background (LGM) (ppb) | 171.0 | 48.8 | 83.7 | 323.6 | 59.2 | 3.1 | 43.8 | 1.5 | 0.5 |
| ICP-SFMS [8] (Holocene) (ppb) | 21.0 |  | 2.3 | - | - | - | 2.0 | 0.03 | 0.02 |
| ICP-SFMS [8] (LGM) (ppb) | 107.6 |  | 79.8 | - | - | - | 57.0 | 1.2 | 0.6 |

Table S5.

Mineral classification scheme based on elemental atomic ratios

| Mineral Name | Representative Formula | Classification Criteria (element atomic ratios)* |
| --- | --- | --- |
| Phyllosilicate (Fe-Mg-Al)-like | (Al,Mg,Fe)_3_(Si,Al)_4_O_10_(OH)_2_ | Si>0; Al>0; (Mg > 0 or Fe > 0); 0.7<(Al + Mg + Fe)/Si<4 |
| Augite-like | (Ca,Na)(Mg,Fe,Al)(Si,Al)_2_O_6_ | Ca, Mg, Fe, Si > 0; 0.6 <(Ca + Mg + Fe)/Si<2.2; 0.275<(Mg + Fe)/Si <1.6; 0.3<Ca/Si<2; |
| Diopside-like | CaMgSi_2_O_6_ | Ca, Mg, Si >0; 0.25<Ca/Si<1; 0.25<Mg/Si<1 |
| Pigeonite-like | (Mg,Fe)_2_Si_2_O_6_ | Mg, Fe, Ca, Si > 0; 0.06 <Ca/Si < 0.25; 0.22<Mg/Si<0.88; 0.22 < Fe/Si < 0.88 |
| High Fe Hornblende-like | Ca_2_(Fe,Mg)_4_Al(Si_7_Al)O_22_(OH)_2_ | Si, Al, Fe, Ca >0; 0.428<(Fe + 2Ti + Al + Mg)/Si<1.5; (2Ca + Na)/Si<1; Mg/Si<0.143; 0.143<Al/Si<0.571; Fe + Al > Mg |
| High Mg Hornblende-like | Ca_2_Mg_4_Al(Si_7_Al)O_22_(OH)_2_ | Si, Al, Fe, Mg, Ca > 0; 0.428 <(Fe + 2Ti + Al + Mg)/Si<3; Fe/Si < 0.143; 0.143<Al/Si<0.571; Mg + Al > Fe |
| Chlorite-like | (Mg,Fe)_5_Al(Si_3_Al)O_10_(OH)_8_ | Si, Al, Fe, Mg > 0; 0.95<(Al + Fe + Mg)/Si<3.6 |
| Kaolinite-like | Al_2_Si_2_O_5_(OH)_4_ | Si, Al > 0; 0.8<Al/Si<1.2 |
| Albite-like | NaAlSi_3_O_8_ | Na, Al, Si > 0; 0.2<Na/Si<0.66; 0.15<Al/Si<1.33; Ca/Si<0.15 |
| Anorthite-like | CaAl_2_Si_2_O_8_ | Ca, Al, Si > 0; 0.25<Ca/Si<1; 0.5<Al/Si<2; Na/Si<0.2 |
| Hypersthene-like | (Mg,Fe)SiO_3_ | Si > 0; (Mg or Fe > 0); 0.75<(Mg + Fe)/Si<1.25; Ca, Al, Na, Ti/Si < 0.1 |
| Quartz-like | SiO_2_ | Si fraction > 0.9 |
| Ca-dominant | CaCO_3_, Ca_5_(PO_4_)_3_(F,Cl,OH), CaF_2_ | Ca fraction > 0.6 |
| Fe-dominant (Hematite/Goethite -like) | Fe_2_O_3_, FeO(OH) | Fe fraction > 0.6 |
| Unclassified | - | Particles not matching any above criteria |

* >0 indicates containing a detectable amount of that element

Table S6. Detection limits (LD) in fg for each isotope and each Taylor Glacier sample measured

| **Sample (Age kyr BP)** | **9.1** | **9.2** | **9.7** | **10** | **10.1** | **10.2** | **10.6** | **10.7** | **11.1** | **11.7** | **12** | **12.2** | **12.6** | **13** | **13.3** |
| --- | --- | --- | --- | --- | --- | --- | --- | --- | --- | --- | --- | --- | --- | --- | --- |
| 23Na | 5.7 | 2.7 | 2.9 | 3.2 | 4 | 2 | 2.5 | 5.3 | 2.7 | 2.9 | 3.2 | 4.2 | 5.2 | 2.3 | 3.7 |
| 24Mg | 2.56 | 1.42 | 1.57 | 1.68 | 1.68 | 1.06 | 1.45 | 2 | 1.38 | 1.55 | 1.76 | 1.99 | 2.64 | 1.31 | 1.77 |
| 27Al | 1.45 | 0.64 | 0.69 | 1.08 | 0.74 | 0.52 | 0.69 | 1.44 | 0.73 | 0.61 | 0.7 | 1 | 1.31 | 0.59 | 0.81 |
| 28Si | 12.4 | 10.7 | 12.9 | 10.3 | 9.8 | 9.6 | 12.6 | 12.5 | 10.5 | 12.4 | 12.2 | 8.6 | 9.1 | 9.4 | 10.4 |
| 44Ca | 11.3 | 15.1 | 13.3 | 6 | 5.5 | 11.2 | 13.1 | 20.1 | 14.7 | 13.1 | 12.3 | 5.4 | 6.2 | 11.3 | 15.4 |
| 45Sc | 0.35 | 0.26 | 0.22 | 0.22 | 0.21 | 0.2 | 0.21 | 0.27 | 0.25 | 0.21 | 0.2 | 0.19 | 0.2 | 0.2 | 0.25 |
| 48Ti | 0.26 | 0.2 | 0.16 | 0.19 | 0.14 | 0.15 | 0.17 | 0.3 | 0.21 | 0.16 | 0.16 | 0.17 | 0.19 | 0.16 | 0.25 |
| 51V | 0.21 | 0.15 | 0.11 | 0.1 | 0.09 | 0.11 | 0.11 | 0.16 | 0.15 | 0.1 | 0.1 | 0.09 | 0.1 | 0.11 | 0.14 |
| 52Cr | 0.58 | 0.39 | 0.31 | 0.32 | 0.32 | 0.3 | 0.31 | 0.37 | 0.38 | 0.31 | 0.31 | 0.32 | 0.32 | 0.29 | 0.38 |
| 54Fe | 3.11 | 4.2 | 3.13 | 3.04 | 2.94 | 2.98 | 3.12 | 4.38 | 4.25 | 3.07 | 3.09 | 2.97 | 3.04 | 2.98 | 4.27 |
| 55Mn | 0.14 | 0.12 | 0.08 | 0.09 | 0.08 | 0.08 | 0.09 | 0.17 | 0.12 | 0.08 | 0.09 | 0.09 | 0.1 | 0.09 | 0.13 |
| 60Ni | 0.61 | 0.51 | 0.38 | 0.38 | 0.38 | 0.37 | 0.38 | 0.65 | 0.51 | 0.38 | 0.38 | 0.38 | 0.39 | 0.37 | 0.51 |
| 59Co | 0.16 | 0.13 | 0.09 | 0.09 | 0.09 | 0.09 | 0.09 | 0.17 | 0.13 | 0.09 | 0.09 | 0.09 | 0.09 | 0.09 | 0.13 |
| 63Cu | 0.26 | 0.23 | 0.17 | 0.19 | 0.15 | 0.17 | 0.17 | 0.89 | 0.22 | 0.17 | 0.16 | 0.16 | 0.19 | 0.15 | 0.23 |
| 64Zn | 0.86 | 0.64 | 0.68 | 0.38 | 0.33 | 0.47 | 0.62 | 0.97 | 0.69 | 0.54 | 0.7 | 0.41 | 0.59 | 0.43 | 0.72 |
| 69Ga | 0.11 | 0.11 | 0.08 | 0.08 | 0.07 | 0.07 | 0.08 | 0.12 | 0.11 | 0.08 | 0.08 | 0.07 | 0.08 | 0.07 | 0.11 |
| 72Ge | 0.56 | 0.56 | 0.42 | 0.34 | 0.34 | 0.41 | 0.42 | 0.57 | 0.56 | 0.42 | 0.42 | 0.34 | 0.34 | 0.41 | 0.56 |
| 75As | 0.59 | 0.63 | 0.45 | 0.41 | 0.4 | 0.44 | 0.45 | 0.61 | 0.62 | 0.45 | 0.44 | 0.41 | 0.41 | 0.44 | 0.62 |
| 78Se | 4.91 | 5.01 | 3.73 | 3.64 | 3.6 | 3.55 | 3.72 | 5.35 | 4.98 | 3.71 | 3.71 | 3.6 | 3.62 | 3.54 | 5 |
| 85Rb | 0.13 | 0.13 | 0.09 | 0.09 | 0.09 | 0.08 | 0.09 | 0.13 | 0.13 | 0.09 | 0.09 | 0.09 | 0.09 | 0.08 | 0.13 |
| 88Sr | 0.1 | 0.1 | 0.07 | 0.07 | 0.07 | 0.07 | 0.07 | 0.11 | 0.1 | 0.07 | 0.08 | 0.07 | 0.08 | 0.08 | 0.12 |
| 89Y | 0.06 | 0.06 | 0.05 | 0.04 | 0.04 | 0.04 | 0.04 | 0.06 | 0.06 | 0.04 | 0.04 | 0.04 | 0.04 | 0.04 | 0.06 |
| 90Zr | 0.12 | 0.13 | 0.09 | 0.08 | 0.08 | 0.09 | 0.09 | 0.14 | 0.13 | 0.09 | 0.09 | 0.08 | 0.09 | 0.09 | 0.13 |
| 93Nb | 0.08 | 0.08 | 0.06 | 0.05 | 0.05 | 0.05 | 0.06 | 0.07 | 0.08 | 0.06 | 0.06 | 0.05 | 0.05 | 0.05 | 0.08 |
| 95Mo | 0.44 | 0.44 | 0.32 | 0.26 | 0.26 | 0.31 | 0.32 | 0.41 | 0.43 | 0.31 | 0.31 | 0.26 | 0.26 | 0.3 | 0.43 |
| 101Ru | 0.5 | 0.42 | 0.31 | 0.21 | 0.21 | 0.28 | 0.3 | 0.36 | 0.4 | 0.29 | 0.28 | 0.21 | 0.21 | 0.27 | 0.39 |
| 103Rh | 0.06 | 0.07 | 0.05 | 0.04 | 0.04 | 0.05 | 0.05 | 0.07 | 0.07 | 0.05 | 0.05 | 0.04 | 0.04 | 0.05 | 0.07 |
| 105Pd | 0.35 | 0.34 | 0.26 | 0.18 | 0.18 | 0.24 | 0.26 | 0.31 | 0.34 | 0.26 | 0.26 | 0.18 | 0.18 | 0.24 | 0.34 |
| 107Ag | 0.17 | 0.24 | 0.19 | 0.09 | 0.09 | 0.19 | 0.19 | 0.24 | 0.23 | 0.19 | 0.19 | 0.09 | 0.09 | 0.19 | 0.23 |
| 111Cd | 0.45 | 0.6 | 0.47 | 0.3 | 0.3 | 0.41 | 0.46 | 0.56 | 0.6 | 0.46 | 0.46 | 0.3 | 0.3 | 0.41 | 0.6 |
| 115In | 0.05 | 0.06 | 0.05 | 0.03 | 0.03 | 0.04 | 0.05 | 0.06 | 0.06 | 0.05 | 0.05 | 0.03 | 0.03 | 0.04 | 0.06 |
| 120Sn | 0.15 | 0.2 | 0.16 | 0.1 | 0.1 | 0.13 | 0.15 | 0.19 | 0.19 | 0.14 | 0.14 | 0.1 | 0.1 | 0.13 | 0.19 |
| 121Sb | 0.16 | 0.22 | 0.17 | 0.11 | 0.11 | 0.15 | 0.17 | 0.19 | 0.22 | 0.17 | 0.17 | 0.11 | 0.11 | 0.15 | 0.21 |
| 130Te | 0.36 | 0.49 | 0.38 | 0.26 | 0.26 | 0.34 | 0.37 | 0.48 | 0.48 | 0.37 | 0.37 | 0.26 | 0.26 | 0.34 | 0.48 |
| 133Cs | 0.05 | 0.06 | 0.05 | 0.04 | 0.04 | 0.04 | 0.04 | 0.05 | 0.06 | 0.04 | 0.04 | 0.04 | 0.04 | 0.04 | 0.06 |
| 138Ba | 0.08 | 0.1 | 0.09 | 0.05 | 0.05 | 0.07 | 0.08 | 0.1 | 0.09 | 0.08 | 0.08 | 0.05 | 0.06 | 0.07 | 0.1 |
| 139La | 0.04 | 0.05 | 0.04 | 0.03 | 0.03 | 0.04 | 0.04 | 0.05 | 0.05 | 0.04 | 0.04 | 0.03 | 0.03 | 0.04 | 0.05 |
| 140Ce | 0.05 | 0.06 | 0.05 | 0.04 | 0.03 | 0.04 | 0.05 | 0.05 | 0.06 | 0.04 | 0.05 | 0.03 | 0.04 | 0.04 | 0.06 |
| 141Pr | 0.04 | 0.05 | 0.04 | 0.02 | 0.02 | 0.03 | 0.04 | 0.04 | 0.05 | 0.04 | 0.04 | 0.02 | 0.02 | 0.03 | 0.05 |
| 143Nd | 0.27 | 0.38 | 0.3 | 0.18 | 0.18 | 0.27 | 0.3 | 0.34 | 0.38 | 0.3 | 0.3 | 0.18 | 0.19 | 0.27 | 0.38 |
| 147Sm | 0.22 | 0.31 | 0.25 | 0.14 | 0.14 | 0.22 | 0.25 | 0.28 | 0.31 | 0.25 | 0.25 | 0.14 | 0.15 | 0.22 | 0.31 |
| 153Eu | 0.06 | 0.09 | 0.07 | 0.04 | 0.04 | 0.06 | 0.07 | 0.08 | 0.08 | 0.07 | 0.07 | 0.04 | 0.04 | 0.06 | 0.08 |
| 157Gd | 0.19 | 0.27 | 0.22 | 0.13 | 0.12 | 0.2 | 0.22 | 0.25 | 0.27 | 0.22 | 0.22 | 0.12 | 0.13 | 0.2 | 0.27 |
| 159Tb | 0.04 | 0.05 | 0.04 | 0.02 | 0.02 | 0.03 | 0.04 | 0.04 | 0.05 | 0.04 | 0.04 | 0.02 | 0.02 | 0.03 | 0.05 |
| 163Dy | 0.13 | 0.19 | 0.15 | 0.08 | 0.08 | 0.14 | 0.15 | 0.17 | 0.19 | 0.15 | 0.15 | 0.08 | 0.08 | 0.14 | 0.19 |
| 165Ho | 0.03 | 0.05 | 0.04 | 0.02 | 0.02 | 0.03 | 0.04 | 0.04 | 0.05 | 0.04 | 0.04 | 0.02 | 0.02 | 0.03 | 0.05 |
| 167Er | 0.14 | 0.22 | 0.17 | 0.09 | 0.09 | 0.15 | 0.17 | 0.19 | 0.21 | 0.17 | 0.17 | 0.09 | 0.09 | 0.15 | 0.21 |
| 169Tm | 0.03 | 0.05 | 0.04 | 0.02 | 0.02 | 0.03 | 0.04 | 0.04 | 0.05 | 0.04 | 0.04 | 0.02 | 0.02 | 0.03 | 0.05 |
| 172Yb | 0.14 | 0.21 | 0.17 | 0.09 | 0.09 | 0.15 | 0.17 | 0.19 | 0.21 | 0.17 | 0.17 | 0.09 | 0.09 | 0.15 | 0.21 |
| 175Lu | 0.03 | 0.05 | 0.04 | 0.02 | 0.02 | 0.04 | 0.04 | 0.04 | 0.05 | 0.04 | 0.04 | 0.02 | 0.02 | 0.04 | 0.05 |
| 178Hf | 0.11 | 0.2 | 0.16 | 0.08 | 0.08 | 0.14 | 0.16 | 0.17 | 0.2 | 0.16 | 0.16 | 0.08 | 0.08 | 0.14 | 0.2 |
| 181Ta | 0.04 | 0.06 | 0.05 | 0.02 | 0.02 | 0.04 | 0.05 | 0.05 | 0.06 | 0.05 | 0.05 | 0.02 | 0.02 | 0.04 | 0.06 |
| 184W | 0.19 | 0.23 | 0.18 | 0.08 | 0.08 | 0.16 | 0.17 | 0.18 | 0.21 | 0.17 | 0.17 | 0.08 | 0.08 | 0.15 | 0.21 |
| 187Re | 0.06 | 0.13 | 0.11 | 0.04 | 0.04 | 0.1 | 0.09 | 0.08 | 0.12 | 0.09 | 0.08 | 0.04 | 0.04 | 0.08 | 0.11 |
| 190Os | 0.16 | 0.55 | 0.46 | 0.21 | 0.2 | 0.42 | 0.33 | 0.23 | 0.45 | 0.29 | 0.27 | 0.19 | 0.2 | 0.32 | 0.38 |
| 193Ir | 0.31 | 0.16 | 0.11 | 0.05 | 0.05 | 0.11 | 0.1 | 0.11 | 0.14 | 0.1 | 0.1 | 0.04 | 0.05 | 0.09 | 0.13 |
| 195Pt | 0.18 | 0.28 | 0.23 | 0.11 | 0.11 | 0.2 | 0.22 | 0.24 | 0.27 | 0.22 | 0.22 | 0.11 | 0.11 | 0.19 | 0.26 |
| 197Au | 0.1 | 0.15 | 0.12 | 0.06 | 0.06 | 0.11 | 0.12 | 0.14 | 0.15 | 0.12 | 0.12 | 0.06 | 0.06 | 0.11 | 0.15 |
| 205Tl | 0.05 | 0.09 | 0.07 | 0.04 | 0.04 | 0.06 | 0.07 | 0.08 | 0.09 | 0.07 | 0.07 | 0.04 | 0.04 | 0.06 | 0.09 |
| 208Pb | 0.1 | 0.15 | 0.13 | 0.06 | 0.05 | 0.12 | 0.13 | 0.15 | 0.15 | 0.13 | 0.13 | 0.06 | 0.07 | 0.12 | 0.15 |
| 209Bi | 0.06 | 0.1 | 0.08 | 0.02 | 0.02 | 0.07 | 0.07 | 0.07 | 0.09 | 0.07 | 0.07 | 0.02 | 0.02 | 0.06 | 0.08 |
| 232Th | 0.04 | 0.07 | 0.06 | 0.02 | 0.02 | 0.06 | 0.06 | 0.06 | 0.07 | 0.06 | 0.06 | 0.02 | 0.02 | 0.06 | 0.07 |
| 238U | 0.04 | 0.08 | 0.07 | 0.02 | 0.02 | 0.06 | 0.06 | 0.07 | 0.08 | 0.06 | 0.06 | 0.02 | 0.03 | 0.06 | 0.08 |

**Table S6. continued**

| **Sample (Age kyr BP)** | **13.5** | **14** | **14.3** | **14.8** | **15.3** | **15.6** | **16** | **17.1** | **21.9** | **26.8** | **32.2** | **34.5** | **44.4** |
| --- | --- | --- | --- | --- | --- | --- | --- | --- | --- | --- | --- | --- | --- |
| 23Na | 7.9 | 3.2 | 6.3 | 2.7 | 4.4 | 7.1 | 3.3 | 5.8 | 10.3 | 2.4 | 9.8 | 6.9 | 4 |
| 24Mg | 3.94 | 1.68 | 2.92 | 1.48 | 2.58 | 4.13 | 2.32 | 4.13 | 3.09 | 1.45 | 4.87 | 3.85 | 2.46 |
| 27Al | 2.21 | 0.66 | 1.37 | 0.85 | 1.39 | 2.61 | 1.86 | 3.9 | 2.4 | 1.12 | 2.78 | 2.17 | 1.28 |
| 28Si | 12.5 | 12.1 | 12.3 | 9.3 | 11.9 | 12.5 | 10.9 | 15.5 | 12.8 | 10.2 | 16.1 | 9.3 | 12.2 |
| 44Ca | 12 | 12.2 | 9 | 11.6 | 22.9 | 10.1 | 16.9 | 24.6 | 9.5 | 15.7 | 16 | 7.3 | 14.1 |
| 45Sc | 0.31 | 0.2 | 0.31 | 0.19 | 0.22 | 0.3 | 0.25 | 0.29 | 0.27 | 0.24 | 0.36 | 0.19 | 0.2 |
| 48Ti | 0.34 | 0.16 | 0.28 | 0.19 | 0.31 | 0.4 | 0.37 | 0.63 | 0.33 | 0.28 | 0.42 | 0.3 | 0.23 |
| 51V | 0.19 | 0.1 | 0.17 | 0.11 | 0.13 | 0.18 | 0.16 | 0.2 | 0.19 | 0.14 | 0.27 | 0.12 | 0.11 |
| 52Cr | 0.59 | 0.31 | 0.57 | 0.29 | 0.36 | 0.57 | 0.38 | 0.51 | 0.59 | 0.38 | 0.59 | 0.32 | 0.31 |
| 54Fe | 3.41 | 3.11 | 3.26 | 3.09 | 4.83 | 3.66 | 5.13 | 7.84 | 3.5 | 4.5 | 3.66 | 3.68 | 3.42 |
| 55Mn | 0.16 | 0.08 | 0.15 | 0.11 | 0.15 | 0.24 | 0.2 | 0.28 | 0.19 | 0.15 | 0.26 | 0.16 | 0.14 |
| 60Ni | 0.63 | 0.37 | 0.61 | 0.37 | 0.46 | 0.7 | 0.52 | 0.6 | 0.66 | 0.52 | 0.63 | 0.4 | 0.38 |
| 59Co | 0.16 | 0.09 | 0.16 | 0.09 | 0.11 | 0.16 | 0.13 | 0.15 | 0.18 | 0.13 | 0.16 | 0.1 | 0.1 |
| 63Cu | 0.3 | 0.16 | 0.24 | 0.17 | 0.22 | 0.26 | 0.23 | 0.42 | 0.28 | 0.23 | 0.29 | 0.18 | 0.17 |
| 64Zn | 1.21 | 0.43 | 0.61 | 0.44 | 1.1 | 0.75 | 0.78 | 0.75 | 0.76 | 0.61 | 1.14 | 0.38 | 0.55 |
| 69Ga | 0.11 | 0.08 | 0.11 | 0.08 | 0.08 | 0.12 | 0.12 | 0.15 | 0.13 | 0.12 | 0.13 | 0.09 | 0.08 |
| 72Ge | 0.53 | 0.42 | 0.52 | 0.41 | 0.36 | 0.52 | 0.57 | 0.58 | 0.53 | 0.57 | 0.58 | 0.34 | 0.42 |
| 75As | 0.57 | 0.44 | 0.54 | 0.43 | 0.4 | 0.55 | 0.62 | 0.63 | 0.6 | 0.62 | 0.65 | 0.42 | 0.44 |
| 78Se | 4.64 | 3.71 | 4.59 | 3.53 | 3.11 | 4.56 | 5 | 5.38 | 5.18 | 5.02 | 4.91 | 3.65 | 3.7 |
| 85Rb | 0.12 | 0.09 | 0.12 | 0.09 | 0.09 | 0.13 | 0.13 | 0.16 | 0.14 | 0.13 | 0.14 | 0.1 | 0.09 |
| 88Sr | 0.12 | 0.08 | 0.11 | 0.08 | 0.09 | 0.13 | 0.14 | 0.18 | 0.15 | 0.11 | 0.17 | 0.12 | 0.11 |
| 89Y | 0.07 | 0.04 | 0.06 | 0.04 | 0.04 | 0.07 | 0.07 | 0.08 | 0.06 | 0.07 | 0.07 | 0.04 | 0.05 |
| 90Zr | 0.13 | 0.09 | 0.12 | 0.09 | 0.08 | 0.13 | 0.15 | 0.17 | 0.14 | 0.14 | 0.13 | 0.1 | 0.1 |
| 93Nb | 0.08 | 0.06 | 0.07 | 0.05 | 0.05 | 0.07 | 0.08 | 0.08 | 0.08 | 0.08 | 0.07 | 0.05 | 0.06 |
| 95Mo | 0.43 | 0.32 | 0.42 | 0.31 | 0.26 | 0.41 | 0.43 | 0.42 | 0.43 | 0.45 | 0.41 | 0.27 | 0.32 |
| 101Ru | 0.44 | 0.28 | 0.43 | 0.27 | 0.21 | 0.43 | 0.38 | 0.36 | 0.44 | 0.37 | 0.41 | 0.21 | 0.28 |
| 103Rh | 0.06 | 0.05 | 0.06 | 0.05 | 0.04 | 0.06 | 0.07 | 0.07 | 0.07 | 0.07 | 0.06 | 0.04 | 0.05 |
| 105Pd | 0.31 | 0.26 | 0.3 | 0.24 | 0.19 | 0.3 | 0.34 | 0.32 | 0.3 | 0.34 | 0.34 | 0.18 | 0.26 |
| 107Ag | 0.12 | 0.19 | 0.11 | 0.19 | 0.09 | 0.11 | 0.23 | 0.24 | 0.13 | 0.24 | 0.19 | 0.09 | 0.19 |
| 111Cd | 0.42 | 0.46 | 0.4 | 0.41 | 0.34 | 0.4 | 0.6 | 0.56 | 0.46 | 0.6 | 0.48 | 0.3 | 0.46 |
| 115In | 0.05 | 0.05 | 0.05 | 0.04 | 0.03 | 0.05 | 0.06 | 0.06 | 0.07 | 0.06 | 0.05 | 0.03 | 0.05 |
| 120Sn | 0.15 | 0.15 | 0.14 | 0.13 | 0.1 | 0.14 | 0.19 | 0.18 | 0.15 | 0.18 | 0.15 | 0.1 | 0.14 |
| 121Sb | 0.17 | 0.17 | 0.16 | 0.15 | 0.11 | 0.16 | 0.22 | 0.19 | 0.17 | 0.22 | 0.17 | 0.11 | 0.17 |
| 130Te | 0.37 | 0.38 | 0.37 | 0.34 | 0.23 | 0.37 | 0.48 | 0.48 | 0.37 | 0.48 | 0.38 | 0.26 | 0.38 |
| 133Cs | 0.05 | 0.04 | 0.05 | 0.04 | 0.03 | 0.05 | 0.06 | 0.06 | 0.06 | 0.06 | 0.05 | 0.04 | 0.05 |
| 138Ba | 0.09 | 0.08 | 0.08 | 0.08 | 0.08 | 0.11 | 0.13 | 0.18 | 0.11 | 0.11 | 0.13 | 0.08 | 0.1 |
| 139La | 0.05 | 0.04 | 0.04 | 0.04 | 0.03 | 0.05 | 0.06 | 0.06 | 0.04 | 0.05 | 0.05 | 0.03 | 0.05 |
| 140Ce | 0.06 | 0.05 | 0.04 | 0.05 | 0.04 | 0.06 | 0.08 | 0.08 | 0.05 | 0.06 | 0.06 | 0.04 | 0.06 |
| 141Pr | 0.05 | 0.04 | 0.03 | 0.03 | 0.02 | 0.04 | 0.05 | 0.05 | 0.04 | 0.05 | 0.04 | 0.02 | 0.04 |
| 143Nd | 0.29 | 0.3 | 0.25 | 0.27 | 0.19 | 0.27 | 0.39 | 0.38 | 0.28 | 0.39 | 0.3 | 0.19 | 0.3 |
| 147Sm | 0.23 | 0.25 | 0.2 | 0.22 | 0.15 | 0.21 | 0.32 | 0.3 | 0.22 | 0.32 | 0.24 | 0.15 | 0.25 |
| 153Eu | 0.07 | 0.07 | 0.05 | 0.06 | 0.04 | 0.06 | 0.09 | 0.08 | 0.06 | 0.08 | 0.07 | 0.04 | 0.07 |
| 157Gd | 0.2 | 0.22 | 0.18 | 0.2 | 0.13 | 0.18 | 0.28 | 0.26 | 0.19 | 0.28 | 0.21 | 0.13 | 0.22 |
| 159Tb | 0.05 | 0.04 | 0.03 | 0.03 | 0.02 | 0.04 | 0.05 | 0.04 | 0.04 | 0.05 | 0.05 | 0.02 | 0.04 |
| 163Dy | 0.14 | 0.15 | 0.12 | 0.14 | 0.09 | 0.12 | 0.2 | 0.18 | 0.13 | 0.19 | 0.14 | 0.08 | 0.15 |
| 165Ho | 0.04 | 0.04 | 0.03 | 0.03 | 0.02 | 0.03 | 0.05 | 0.04 | 0.03 | 0.05 | 0.04 | 0.02 | 0.04 |
| 167Er | 0.15 | 0.17 | 0.13 | 0.15 | 0.09 | 0.13 | 0.22 | 0.2 | 0.14 | 0.22 | 0.15 | 0.09 | 0.17 |
| 169Tm | 0.04 | 0.04 | 0.03 | 0.03 | 0.02 | 0.03 | 0.05 | 0.04 | 0.03 | 0.05 | 0.04 | 0.02 | 0.04 |
| 172Yb | 0.14 | 0.17 | 0.13 | 0.15 | 0.09 | 0.13 | 0.22 | 0.2 | 0.14 | 0.21 | 0.15 | 0.09 | 0.17 |
| 175Lu | 0.04 | 0.04 | 0.03 | 0.04 | 0.02 | 0.03 | 0.05 | 0.05 | 0.03 | 0.05 | 0.04 | 0.02 | 0.04 |
| 178Hf | 0.12 | 0.16 | 0.12 | 0.14 | 0.08 | 0.12 | 0.2 | 0.18 | 0.12 | 0.2 | 0.13 | 0.08 | 0.16 |
| 181Ta | 0.04 | 0.05 | 0.04 | 0.04 | 0.03 | 0.04 | 0.06 | 0.05 | 0.04 | 0.06 | 0.04 | 0.02 | 0.05 |
| 184W | 0.16 | 0.17 | 0.14 | 0.15 | 0.09 | 0.13 | 0.21 | 0.18 | 0.14 | 0.2 | 0.15 | 0.08 | 0.16 |
| 187Re | 0.06 | 0.08 | 0.06 | 0.07 | 0.04 | 0.06 | 0.1 | 0.08 | 0.06 | 0.1 | 0.06 | 0.04 | 0.08 |
| 190Os | 0.15 | 0.25 | 0.14 | 0.25 | 0.1 | 0.14 | 0.35 | 0.23 | 0.15 | 0.31 | 0.15 | 0.2 | 0.25 |
| 193Ir | 0.2 | 0.1 | 0.17 | 0.09 | 0.06 | 0.15 | 0.13 | 0.1 | 0.16 | 0.12 | 0.14 | 0.05 | 0.1 |
| 195Pt | 0.18 | 0.22 | 0.17 | 0.19 | 0.11 | 0.17 | 0.26 | 0.24 | 0.18 | 0.26 | 0.18 | 0.11 | 0.22 |
| 197Au | 0.11 | 0.12 | 0.1 | 0.1 | 0.06 | 0.1 | 0.15 | 0.14 | 0.1 | 0.15 | 0.1 | 0.06 | 0.12 |
| 205Tl | 0.05 | 0.07 | 0.05 | 0.06 | 0.04 | 0.05 | 0.09 | 0.08 | 0.06 | 0.11 | 0.06 | 0.04 | 0.07 |
| 208Pb | 0.11 | 0.13 | 0.09 | 0.12 | 0.09 | 0.1 | 0.16 | 0.15 | 0.11 | 0.15 | 0.12 | 0.07 | 0.13 |
| 209Bi | 0.08 | 0.07 | 0.08 | 0.06 | 0.04 | 0.09 | 0.08 | 0.07 | 0.06 | 0.08 | 0.09 | 0.03 | 0.07 |
| 232Th | 0.04 | 0.06 | 0.04 | 0.06 | 0.03 | 0.04 | 0.08 | 0.07 | 0.04 | 0.08 | 0.05 | 0.03 | 0.07 |
| 238U | 0.05 | 0.06 | 0.04 | 0.06 | 0.03 | 0.04 | 0.08 | 0.07 | 0.04 | 0.08 | 0.05 | 0.03 | 0.07 |
